# Effects of carbohydrate, caffeine and menthol mouth rinses on exercise performance and ratings of perceived exertion: a systematic review and network meta-analysis

**DOI:** 10.3389/fnut.2026.1877453

**Published:** 2026-08-19

**Authors:** Qing Li, Shichao Liu, Cheng Miao, Yi Zhang, Yunhang Lu

**Affiliations:** 1 School of Physical Education and Sports Science, Soochow University, Suzhou, China; 2 School of General Education, Zhengzhou Institute of Food Technology, Zhengzhou, China; 3 Department of Physical Education, Kyungpook National University, Daegu, Republic of Korea

**Keywords:** caffeine mouth rinse, carbohydrate mouth rinse, exercise performance, menthol mouth rinse, network meta-analysis, ratings of perceived exertion

## Abstract

**Background:**

Mouth rinsing with carbohydrate (CHO), caffeine (CAF), or menthol (MEN) is a non-ingestive strategy that may enhance exercise performance and alter ratings of perceived exertion (RPE), but findings remain inconsistent. This systematic review and network meta-analysis (NMA) compared their relative effects.

**Methods:**

This prospectively registered review (PROSPERO CRD420261282363) followed PRISMA-NMA. PubMed, Embase, Web of Science, the Cochrane Library, and SPORTDiscus were searched to 3 April 2026. Randomized controlled and crossover trials were eligible. RoB 2 and Confidence in Network Meta-Analysis (CINeMA) were used to assess risk of bias and certainty of evidence. Pairwise and network meta-analyses were performed in Stata 18.0, effects were expressed as standardized mean differences (SMDs), and rankings were based on the surface under the cumulative ranking curve (SUCRA).

**Results:**

Fifty-seven studies involving 609 participants were included. The evidence base primarily comprised trained or physically active individuals, and the exercise protocols predominantly involved cycling- or running-based endurance tasks. CAF ranked first for time to exhaustion (SUCRA = 85.1%), work done during a fixed-time test (83.4%), and RPE (74.0%). MEN ranked first for time to complete a fixed amount of work (79.8%) and mean power (77.3%), whereas CHO ranked second across all five outcomes. The NMA showed no statistically significant differences among CHO, CAF, and MEN. Compared with placebo (PLA), CAF (SMD = 0.57, 95% CI: 0.13, 1.01) and CHO (SMD = 0.43, 95% CI: 0.11, 0.75) significantly prolonged time to exhaustion. CAF (SMD = 0.48, 95% CI: 0.11, 0.86) and CHO (SMD = 0.36, 95% CI: 0.12, 0.60) also significantly increased work done during a fixed-time test. CHO significantly reduced RPE (SMD = −0.14, 95% CI: −0.26, −0.02). No significant effects relative to PLA were observed for time to complete a fixed amount of work or mean power.

**Conclusion:**

The relative advantages appeared outcome-specific. CAF showed the most favorable ranking trends for fatigue tolerance, sustained output, and RPE, whereas MEN ranked highest for completion efficiency and mean power. Because CHO, CAF, and MEN did not differ significantly, these rankings should be interpreted probabilistically rather than as evidence of clear superiority.

**Systematic review registration:**

https://www.crd.york.ac.uk/PROSPERO/view/CRD420261282363, identifier CRD420261282363.

## 1 Introduction

Exercise supplement mouth rinse is a non-ingestive nutritional intervention designed to enhance immediate exercise performance. It generally refers to briefly rinsing the mouth with a solution containing functional ingredients before or during exercise and then spitting it out rather than swallowing it (1). This strategy has been widely studied in endurance sports such as cycling and running and has shown certain practical potential (2, 3). Compared with traditional ingestion, mouth rinsing can help avoid the additional splanchnic blood flow demand associated with supplement ingestion and subsequent digestion and absorption, thereby reducing competition for blood flow between the splanchnic circulation and working skeletal muscle during high-intensity exercise while also reducing the gastrointestinal burden associated with supplement intake (4, 5). In recent years, as studies on mouth rinses containing different ingredients such as carbohydrate (CHO), caffeine (CAF), and menthol (MEN) have continued to accumulate, mouth rinsing as a non-ingestive ergogenic strategy has received increasing attention in sports nutrition and exercise performance research (6).

**FIGURE 1:**
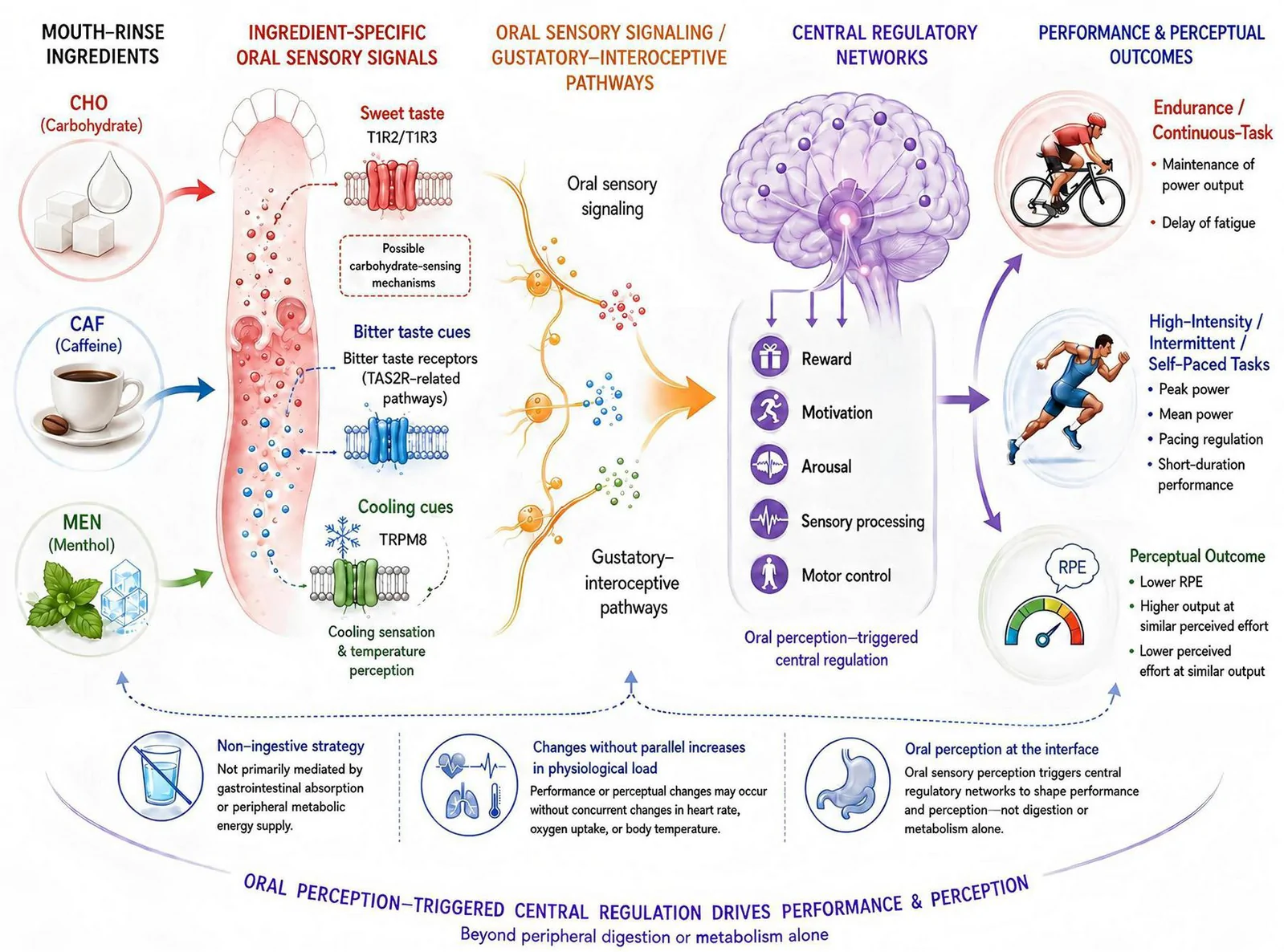
Proposed theoretical mechanism by which carbohydrate, caffeine, and menthol mouth rinses may influence exercise performance and ratings of perceived exertion through oral sensory pathways.

In terms of the underlying mechanisms, different mouth-rinse ingredients may generate distinct sensory stimuli in the oral cavity, and the resulting signals may be transmitted to the central nervous system through corresponding sensory pathways. Specifically, CHO is mainly related to sweet taste and carbohydrate-sensing pathways, including T1R2/T1R3 sweet taste receptors and possible non-sweet carbohydrate-sensing mechanisms (7). CAF primarily provides bitter taste cues that may be perceived through bitter taste receptors and TAS2R-related pathways (8). MEN mainly provides cooling cues, which may be mediated by the TRPM8 cold-sensitive ion channel and related changes in cooling sensation and temperature perception (9). These oral sensory signals may influence central networks associated with reward, motivation, arousal, sensory processing, and motor control through gustatory–interoceptive pathways. Because CHO, CAF, and MEN may activate different oral sensory inputs and subsequent central regulatory processes, their ergogenic effects may vary across exercise tasks and outcome measures. In endurance or continuous tasks, these effects may manifest as the maintenance of power output and delayed fatigue (10, 11). In high-intensity, intermittent, or self-paced tasks, they may involve improvements in peak power, mean power, pacing regulation, or short-duration exercise performance (12, 13). At the same time, mouth-rinse interventions may also affect ratings of perceived exertion (RPE), allowing individuals to maintain higher exercise output at a similar subjective effort level or to perceive lower effort under similar exercise output conditions (14, 15). Notably, these changes in exercise performance or subjective perception are not always accompanied by parallel changes in peripheral physiological load indicators, such as heart rate, oxygen uptake, or body temperature (16–18). Therefore, the dissociation among exercise output, subjective perception, and physiological responses further suggests that the mechanism of mouth-rinse interventions is more consistent with oral perception-triggered central regulation than with peripheral digestion and metabolism alone. Based on these mechanisms, exercise supplement mouth-rinse strategies have become an important research direction in exercise science in recent years, and the intervention has gradually expanded from an early focus on CHO to other ingredients such as CAF and MEN (19–21). To provide a more intuitive overview of how different mouth-rinse ingredients may influence exercise performance and RPE through oral sensory pathways, the proposed theoretical mechanism is summarized in Figure 1.

### 1.1 Evidence from empirical studies

A growing number of empirical studies have evaluated the effects of different mouth-rinse ingredients on exercise performance, but the findings have been inconsistent. For CHO mouth rinse, Brietzke et al. (22) found that it attenuated the decline in exercise performance caused by mental fatigue. However, Pires et al. (23) observed no improvement in performance, RPE, or central fatigue indicators during an incremental test to exhaustion, suggesting that its effects may not appear consistently across all tasks. CAF mouth rinse has also shown substantial variability in outcomes. Karami et al. (24) found that repeated coffee mouth rinsing improved sport-specific endurance and jumping performance in young male five-a-side soccer players, whereas Pataky et al. (25) reported that responses to CAF mouth rinse during a 3-km cycling time trial might vary according to CYP1A2 genotype and time of day. By contrast, research on MEN mouth rinse has focused more on hot environments. Crosby et al. (17) reported that MEN mouth rinse helped maintain relative power output in the heat, but its advantage was not consistently observed under all task conditions. These findings indicate that different mouth-rinse ingredients may vary in their potential mechanisms and in the exercise contexts in which they are most likely to be beneficial. However, individual primary studies alone cannot directly establish the relative effects of different mouth-rinse strategies.

### 1.2 Evidence from previous systematic reviews and meta-analyses

To further integrate these scattered findings from primary studies, previous researchers have conducted systematic reviews and meta-analyses on the effects of single-ingredient mouth rinses. The evidence base, outcomes of interest, and main findings of previous systematic reviews and meta-analyses are summarized in Table 1. In terms of CHO, Hartley et al. (26) showed in a meta-analysis of 35 randomized controlled trials that CHO mouth rinse could improve various types of exercise performance to some extent, including cycling and running. For specific exercise tasks, Brietzke et al. (27) analyzed 13 studies involving 16 experiments and reported that, in cycling time trials using mean power output and completion time as outcomes, CHO mouth rinse significantly increased mean power output but did not significantly affect completion time. In resistance exercise, Oliveira-Silva et al. (28) found that CHO mouth rinse did not significantly improve maximal strength but enhanced muscular endurance performance in healthy adults. Beyond CHO, systematic reviews have also synthesized the effects of CAF and MEN on exercise performance. A systematic review by da Silva et al. (29) suggested that CAF mouth rinse has some potential to improve physical and cognitive performance, but the findings across studies were inconsistent. A recent meta-analytic review from 2026, which included 31 studies and used a three-level meta-analytic approach, further updated the evidence regarding the effects of CAF mouth rinse on exercise and cognitive performance. The results showed only a minimal benefit for exercise performance, which was more likely to be observed in aerobic endurance tasks, in the fed state, and with shorter rinse durations, whereas the cognitive effects were generally inconsistent (30). For exercise performance in hot environments, Gavel et al. (18) reported in a systematic review and meta-analysis that MEN mouth rinse did not produce a significant overall improvement in exercise capacity or performance, although potential benefits may be observed in endurance exercise.

**TABLE 1:** Systematic reviews and meta-analyses of different exercise supplement mouth-rinse interventions.

Study	Ingredient	Review type and evidence base	Exercise context/outcomes	Main findings
Hartley et al. (26)	CHO	Systematic review and meta-analysis; 35 articles included, of which 34 were included in the meta-analysis	Various exercise modes; overall exercise performance	Conventional meta-analysis showed that CHO mouth rinse produced a small improvement in exercise performance, while the robust adjusted model still suggested a potential beneficial trend, although the effect was small.
Brietzke et al. (27)	CHO	Systematic review and meta-analysis; 13 studies and 16 trials included	Cycling time trial; mean power output and completion time	CHO mouth rinse significantly increased mean power output but did not significantly improve time to complete the time trial.
Oliveira-Silva et al. (28)	CHO	Systematic review and meta-analysis; 13 randomized clinical trials included	Maximal strength and muscular endurance	CHO mouth rinse had no significant effect on maximal strength but significantly improved muscular endurance performance.
da Silva et al. (29)	CAF	Systematic review; 18 studies included, of which 15 examined physical performance and 3 examined cognitive performance	Physical performance and cognitive performance	The effects of CAF mouth rinse on physical performance were mixed, whereas cognitive performance showed some positive signals.
Deng et al. (30)	CAF	Meta-analytic review; 31 studies and 167 effect sizes included	Exercise performance and cognitive performance	CAF mouth rinse showed trivial-to-small effects on exercise performance, with benefits more likely in aerobic endurance tasks, fed-state conditions, and shorter rinse durations; cognitive effects were generally unstable.
Gavel et al. (18)	MEN	Systematic review and meta-analysis; 10 MEN mouth-rinse studies included	Exercise capacity and exercise performance, mainly in heat-related/endurance contexts	MEN mouth rinse did not significantly improve overall exercise capacity or performance, although potential benefits may be observed in endurance exercise contexts.

### 1.3 Need for a cross-ingredient network meta-analysis

Overall, previous systematic reviews and traditional pairwise meta-analyses have provided some evidence for the potential effects of single-ingredient mouth rinses, but the current evidence base still has important limitations. Most meta-analyses have examined only one ingredient versus placebo and have lacked direct or indirect comparisons among different mouth-rinse ingredients. This evidence gap creates a practical problem. When multiple mouth-rinse ingredients are available, coaches and sports nutrition professionals still lack clear evidence to determine which ingredient should be prioritized or which is more likely to provide practical benefits across different exercise contexts or task types. Therefore, a unified comparison of different mouth-rinse ingredients is needed rather than continuing to evaluate each ingredient separately against placebo.

On this basis, the present study employed network meta-analysis (NMA) to compare CHO, CAF, MEN, and placebo within a unified analytical framework and to evaluate their relative effects on exercise performance and ratings of perceived exertion among athletes and other trained or physically active individuals (31). By providing cross-ingredient comparative evidence, this study aimed to better inform coaches, athletes, and physically active individuals in the interpretation and selection of mouth-rinse strategies across different exercise contexts and task types.

## 2 Materials and methods

This study was prospectively registered in the International Prospective Register of Systematic Reviews (PROSPERO; registration No. CRD420261282363). The systematic review and NMA were conducted and reported in strict accordance with the Preferred Reporting Items for Systematic Reviews and Meta-Analyses (PRISMA) guidelines (32). The PRISMA-NMA checklist is provided in Supplementary file 1:1.

### 2.1 Inclusion criteria

This study was designed and conducted according to the Participants, Interventions, Comparators, Outcomes, and Study design (PICOS) framework in evidence-based medicine (33).^1^ The inclusion criteria were as follows: (1) Participants: healthy individuals, including adolescent or adult athletes, trained individuals, or physically active individuals, regardless of sport, training level, or sex. (2) Interventions: the experimental group received an oral mouth-rinse intervention with CHO, CAF, or MEN, and participants were required to briefly rinse before or during exercise and spit the solution out rather than swallow it. If a single study included multiple eligible mouth-rinse intervention groups, relevant data were extracted separately when necessary and then handled according to the statistical analysis strategy to avoid double-counting shared control groups. If a study also included other intervention forms unrelated to the present study, then only the groups relevant to the mouth-rinse intervention were included. (3) Comparators: the control condition could include a placebo mouth rinse or water, provided that it offered a valid basis for comparison and that baseline characteristics were comparable between groups. (4) Outcomes: studies had to report at least one outcome related to exercise performance or subjective perception. Exercise performance outcomes included time to exhaustion, work done during a fixed-time test, time to complete a fixed amount of work, and mean power, whereas subjective outcomes included quantifiable indices such as rating of perceived exertion (RPE). The results had to yield raw data that could be extracted or converted for effect-size analysis. (5) Study design: RCTs or randomized crossover trials (crossover RCTs) were included. Studies with a valid comparison between mouth-rinse and control conditions and sufficient outcome data were eligible. If the process of random allocation was not clearly described, eligibility was determined based on the methodological information reported in the original article and then reflected in the subsequent risk-of-bias assessment. Studies using nonrandom allocation methods, such as investigator assignment or participant self-selection, were excluded.

### 2.2 Exclusion criteria

The exclusion criteria were as follows: (1) Participants: studies involving patients, injured populations, special clinical populations, or animals were excluded. Studies that did not report basic demographic characteristics or training background and for which it was impossible to determine whether the participants fell within the scope of healthy athletes or physically active individuals were also excluded. (2) Interventions: studies using ingestion rather than oral mouth rinse were excluded; studies combining other active ingredients when the effects of CHO, CAF, or MEN mouth rinse could not be extracted separately were excluded; and studies that did not clearly report the mouth-rinse ingredient, concentration, duration, timing, or frequency were excluded. (3) Comparators: studies without a placebo or control group were excluded. If the control group was clearly not comparable to the intervention group at baseline, or if the control condition could not serve as a valid basis for comparison, the study was not included. (4) Outcomes: studies were excluded if they did not report outcomes related to exercise performance or subjective perception, if they failed to provide post-intervention or pre-post comparison results, or if the outcome data were incomplete and effect sizes could not be extracted or converted. (5) Study design: single-arm studies without a control group, cohort studies, cross-sectional studies, case series, and other observational studies were excluded, as were non-RCTs that used nonrandom allocation methods such as investigator judgment or participant preference. In addition, gray literature (e.g., conference abstracts and theses), reviews, commentaries, case reports, and studies for which the full text could not be obtained were excluded. Studies not published in English were excluded.

### 2.3 Search strategy

This network meta-analysis followed the PRISMA-NMA guidelines. PubMed, Embase, Web of Science, the Cochrane Library, and SPORTDiscus were systematically searched from database inception to April 3, 2026, with the search restricted to English-language publications. The search strategy combined MeSH terms and free-text terms and focused on three major themes: oral mouth rinse, CHO/CAF/MEN, and exercise performance. Boolean operators (OR/AND) were used to construct the search logic. The complete search strategy is provided in Supplementary file 1:2. In addition, the reference lists of relevant original studies and reviews were manually screened to identify potentially missing records. After duplicate removal, all records were screened in two stages, title/abstract screening and full-text assessment, to determine the final included studies.

### 2.4 Study selection

EndNote 21 was used to remove duplicate records from all retrieved references. Two reviewers (ZY and LQ) independently screened the studies according to the predefined inclusion and exclusion criteria. During the initial screening stage, the two reviewers independently assessed titles and abstracts, excluded clearly ineligible records, and briefly recorded the reasons for exclusion. Of the 57 included studies, 31 were available as open-access articles, whereas the remaining 26 were accessed through university library subscriptions or resources provided by collaborating institutions. Any disagreements were resolved through discussion between the two reviewers. If consensus could not be reached, the issue was escalated to a third reviewer (LY) for adjudication and a final decision.

### 2.5 Data extraction

The following information was extracted from each included study: (1) article title, (2) first author, (3) year of publication, (4) country in which the study was conducted, (5) study type, (6) sample size, (7) sex distribution, (8) mean age, (9) exercise type, (10) outcome indicators, (11) intervention type, (12) concentration, (13) testing method, (14) intervention group’s sample size, (15) intervention group’s mean, (16) intervention group’s standard deviation, (17) control group’s sample size, (18) control group’s mean, (19) control group’s standard deviation, and (20) rinse duration. Participant training status, pre-exercise feeding status, rinse timing, and rinsing frequency were included among the participant and intervention characteristics extracted from each study. All data were recorded in an electronic spreadsheet and independently extracted and cross-checked by two reviewers to ensure accuracy. Any disagreements were resolved through discussion; if consensus could not be reached, a third reviewer made the final decision. For studies with missing key information, the corresponding authors were contacted by email to request additional data. In three studies, the required numerical outcome data were reported only in graphical form, and none of the corresponding authors responded (0/3). Therefore, two reviewers independently digitized the relevant values from the published figures using WebPlotDigitizer (version 5.2; Ankit Rohatgi, United States). Each reviewer independently calibrated the figure axes and extracted the corresponding values. Any discrepancies were resolved by re-examining the axis calibration and data-point locations, repeating the extraction when necessary, and reaching consensus through discussion. If consensus could not be reached, a third reviewer examined the original figures and made the final decision.

### 2.6 Risk of bias assessment and CINeMA

Risk of bias was assessed using the Cochrane Risk of Bias 2 (RoB 2) tool (34). Although both parallel-group randomized controlled trials and randomized crossover trials were eligible according to the inclusion criteria, the final evidence base consisted exclusively of randomized crossover trials; therefore, the RoB 2 version for randomized crossover trials was used. In the RoB 2 assessment, the target effect was defined as the effect of participants being assigned to each mouth-rinse condition. Because objective performance outcomes and self-reported perceptual outcomes may be affected by different sources of bias, risk of bias was assessed at the outcome level rather than assigning a single overall judgment to each study. Accordingly, we assessed objective exercise performance outcomes, including time to exhaustion, work done during a fixed-time test, time to complete a fixed amount of work, and mean power output, separately from the subjective outcome of rating of perceived exertion (RPE). The evaluated domains included bias arising from the randomization process, bias arising from period and carryover effects, bias due to deviations from intended interventions, bias due to missing outcome data, bias in measurement of the outcome, and bias in selection of the reported result. During the assessment, particular attention was given to whether the sensory characteristics of the mouth-rinse solutions, such as menthol-related cooling sensation or caffeine-related bitterness, might compromise blinding or influence participants’ responses. For RPE, incomplete blinding could directly influence self-reported perceptions through expectancy or awareness of the assigned condition. For objectively measured performance outcomes, objective recording was not considered sufficient to eliminate the potential influence of incomplete blinding, because sensory cues could produce expectancy-related changes in exercise effort, pacing strategy, willingness to continue exercise, or power-output regulation. These issues were considered when assessing bias in measurement of the outcome and bias due to deviations from intended interventions, as appropriate. Each domain and the overall risk of bias were judged as “low risk,” “some concerns,” or “high risk.” Two reviewers independently assessed the risk of bias, and disagreements were resolved through discussion with a third reviewer.

The Confidence in Network Meta-Analysis (CINeMA) web application was used to systematically assess the certainty of evidence for the NMA on the basis of the GRADE consistency framework (35, 36). For each treatment comparison and outcome, six domains were evaluated: within-study bias, reporting bias, indirectness, imprecision, heterogeneity, and inconsistency (35). Publication bias was judged using funnel plots combined with Egger’s regression test (37). When a comparison included a small number of studies or a small-study effect was suspected, reporting bias was rated at least as “some concerns.” For imprecision, a standardized mean difference (SMD) of 0.20 was prespecified as the threshold for a minimal important effect. Certainty was downgraded when the 95% confidence interval crossed this threshold or included values compatible with both trivial and potentially important effects (38). Following the CINeMA guidance, the initial confidence for each comparison was assumed to be high. When “some concerns” or “major concerns” were identified in one or more domains, certainty was reduced to moderate, low, or very low (35).

### 2.7 Statistical analysis

Statistical analyses were performed using Stata 18.0. First, conventional pairwise meta-analyses were conducted for comparisons involving at least two eligible studies (39) to evaluate the direct effects of each mouth-rinse intervention relative to placebo on time to exhaustion, work done during a fixed-time test, time to complete a fixed amount of work, mean power, and RPE. Effect sizes were expressed as Hedges’ g and were calculated from the sample size and condition-specific means and standard deviations (SDs). Because the final evidence base consisted exclusively of randomized crossover trials, the within-participant correlation was taken into account when estimating the variance and standard error of each effect size. As exact paired statistics and within-participant correlation coefficients were generally unavailable, a correlation coefficient of *r* = 0.50 was assumed. This approach followed the Cochrane Handbook guidance to account for within-participant correlation in crossover trials. The assumed correlation coefficient was incorporated into the calculation of the variance, standard error, and 95% confidence interval (CI) of Hedges’ g. To evaluate the robustness of the findings to this assumption, sensitivity analyses were conducted using alternative within-participant correlation coefficients of *r* = 0.30 and *r* = 0.70, which were selected symmetrically around the primary assumption to represent lower and higher within-participant correlations. For each alternative coefficient, the effect-size variances, standard errors, and 95% CIs were recalculated, and both the pairwise and network meta-analyses were repeated for all outcomes. SUCRA rankings were also recalculated to assess the stability of the intervention rankings. All pairwise analyses used random-effects models (40). For pairwise meta-analyses, when multiple concentration-specific arms of the same mouth-rinse ingredient shared the same control group within a study, these arms were combined into a single comparison before effect-size calculation. When an article reported two or more independent experiments with separate intervention and control conditions, these experiments were retained as separate comparisons. In the network meta-analysis, different concentrations of the same mouth-rinse ingredient were combined within the same intervention node according to ingredient category (e.g., CHO, CAF, MEN, or PLA) to preserve the network structure and enable cross-ingredient comparisons. Therefore, the number of pairwise comparisons reported in Supplementary file 2 may exceed the number of unique studies reported in the main text. According to Cohen’s conventional benchmarks, SMD values of approximately 0.20, 0.50, and 0.80 were interpreted as small, medium, and large effects, respectively (33). Heterogeneity was assessed using the I^2^ statistic and Cochran’s Q test, with *I*^2^ < 25% indicating very low heterogeneity, 25–50% denoting low heterogeneity, 50–75% reflecting moderate heterogeneity, and ≥ 75% signifying high heterogeneity (33). Exploratory random-effects meta-regression analyses were conducted only for comparisons including at least 10 studies, in accordance with the recommendations of the Cochrane Handbook, to examine whether participants’ mean age, study sample size, and rinse duration were associated with effect estimates. Each covariate was examined in a separate model. These analyses were considered exploratory and were used only to identify potential sources of heterogeneity rather than as confirmatory evidence.

On this basis, Stata’s network command series was used for the NMA, integrating direct and indirect evidence to evaluate the relative effects of CHO, CAF, MEN, and placebo (PLA) across different outcomes (41). In the network, PLA was used as a common label for placebo-related control conditions, including placebo mouth rinse and water, when applicable. First, network plots were generated to visually represent the comparative structure of interventions, with node size weighted by the number of participants and line thickness proportional to the number of direct comparisons (42). Network consistency was assessed according to the network structure. When closed loops were present, global inconsistency was tested, and local inconsistency was assessed using node-splitting. When no closed loops were present, formal inconsistency testing was not feasible, and a consistency model was used. The surface under the cumulative ranking curve (SUCRA) was calculated for each intervention and used to comprehensively evaluate and rank the different mouth-rinse strategies. To facilitate a comparison of results, we generated forest plots and cumulative ranking plots, and league tables were used to summarize all pairwise comparison results, thereby systematically presenting the relative effects and ranking probabilities of the interventions across different outcomes (43, 44). In addition, we invited a statistical methodologist with experience in biostatistics and evidence synthesis to review the statistical analysis plan, data structure, and interpretation of the pairwise and network meta-analysis results, thereby enhancing the standardization of the statistical procedures and the reliability of result interpretation.

### 2.8 Publication bias

Potential publication bias was visually assessed by generating comparison-adjusted funnel plots and further quantified using Egger’s regression test (37). When the test indicated possible publication bias, the trim-and-fill method was used to evaluate its potential influence on the overall effect estimate (45).

## 3 Results

### 3.1 Study selection and characteristics

**FIGURE 2:**
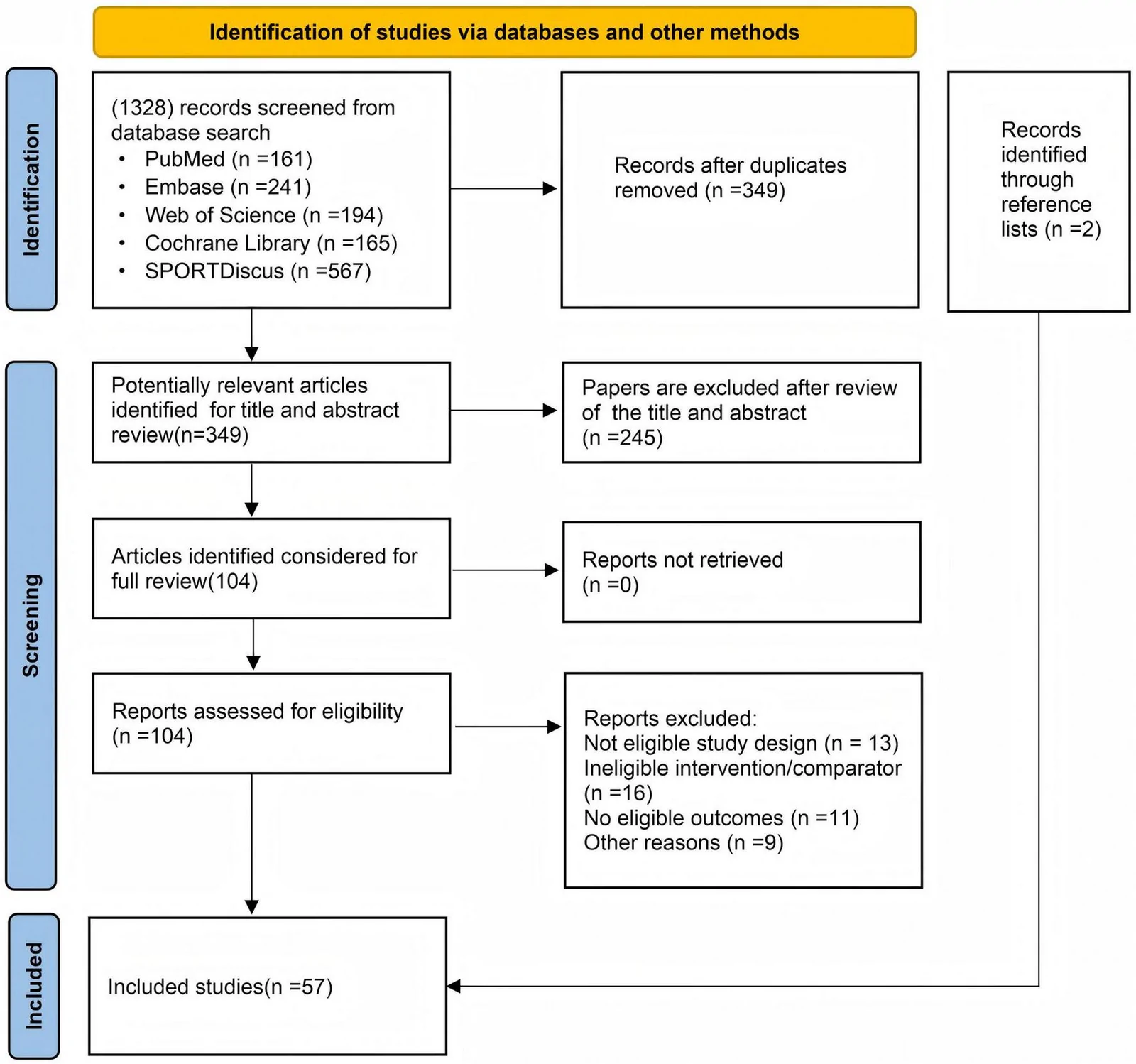
Flow diagram of study selection.

The electronic database search identified 1,328 records, and two additional records were identified through reference-list tracking. After duplicate removal, 349 records from database searching remained for title and abstract screening. Of these, 245 records were excluded, leaving 104 full-text articles for eligibility assessment. After full-text assessment, 49 articles were excluded, and 55 studies were retained from database searching. The two records identified through reference-list tracking were also eligible and were added to the database-derived studies. Finally, 57 studies involving 609 participants were included in the systematic review and network meta-analysis (the selection process is shown in Figure 2). When classified by mouth-rinse ingredient, 37 studies involved carbohydrate mouth rinse, 15 involved caffeine mouth rinse, and 9 involved menthol mouth rinse. It should be noted that four studies included both CHO and CAF intervention conditions; therefore, the ingredient-specific study counts were not mutually exclusive and may exceed the total number of included studies.

These studies included a total of 609 participants, comprising healthy adolescent or adult athletes, trained individuals, or physically active individuals. The exercise protocols predominantly involved endurance tasks using cycling or running. The studies were conducted across a wide range of countries, including the United Kingdom, Brazil, Canada, Portugal, and Lebanon. The main outcomes assessed were time to exhaustion, work done during a fixed-time test, time to complete a fixed amount of work, mean power output, and RPE. The included interventions primarily involved CHO, CAF, and MEN mouth rinses compared with PLA. All included studies were randomized crossover trials. Regarding rinse duration, 26 studies used a 5-s rinse only, 28 used a 10-s rinse only, one directly compared 5- and 10-s rinsing, and two used other durations of 8 or 15 s. Thus, 55 of the 57 studies used a rinse duration of either 5 or 10 s. Eleven studies used a single-rinse protocol, whereas 46 used repeated rinsing. Among the repeated-rinse protocols, rinsing frequency varied according to the exercise task and was commonly determined by predetermined time intervals, distance or proportion of work completed, or specific stages of the exercise protocol; only one study explicitly used an every-5-km rinsing schedule. For CHO mouth rinses, 6.4% was the most frequently investigated concentration and was used in 18 of the 37 CHO studies. For CAF, 1.2% was the most common concentration and was examined in 6 of the 15 studies. For MEN, 0.01% was the predominant concentration and was used in 7 of the 9 studies. The remaining studies used other concentrations or compared multiple concentrations within the same trial. Regarding pre-exercise feeding status, 10 studies were conducted exclusively under fed conditions, 18 exclusively under fasted conditions, four included both fed and fasted conditions, and 25 did not clearly report or standardize feeding status. Detailed participant and intervention characteristics are presented in Supplementary file 1:3.

### 3.2 Risk of bias assessment and CINeMA

**FIGURE 3:**
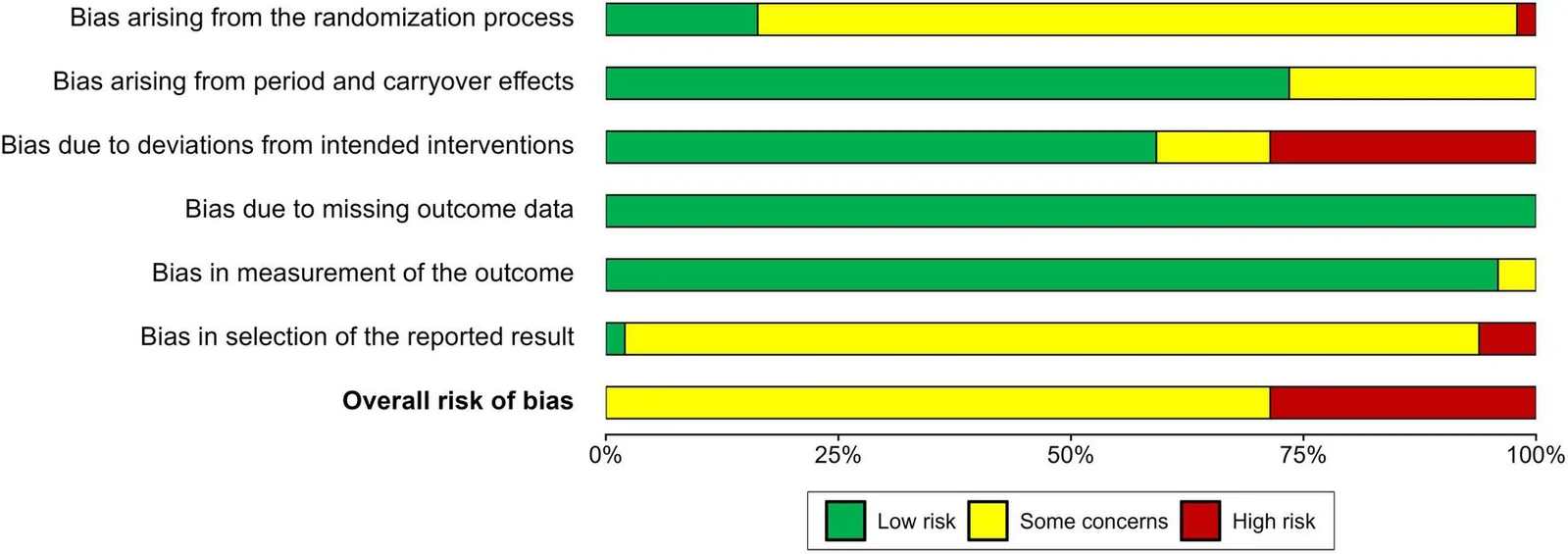
Summary risk-of-bias assessment for objective exercise performance outcomes.

**FIGURE 4:**
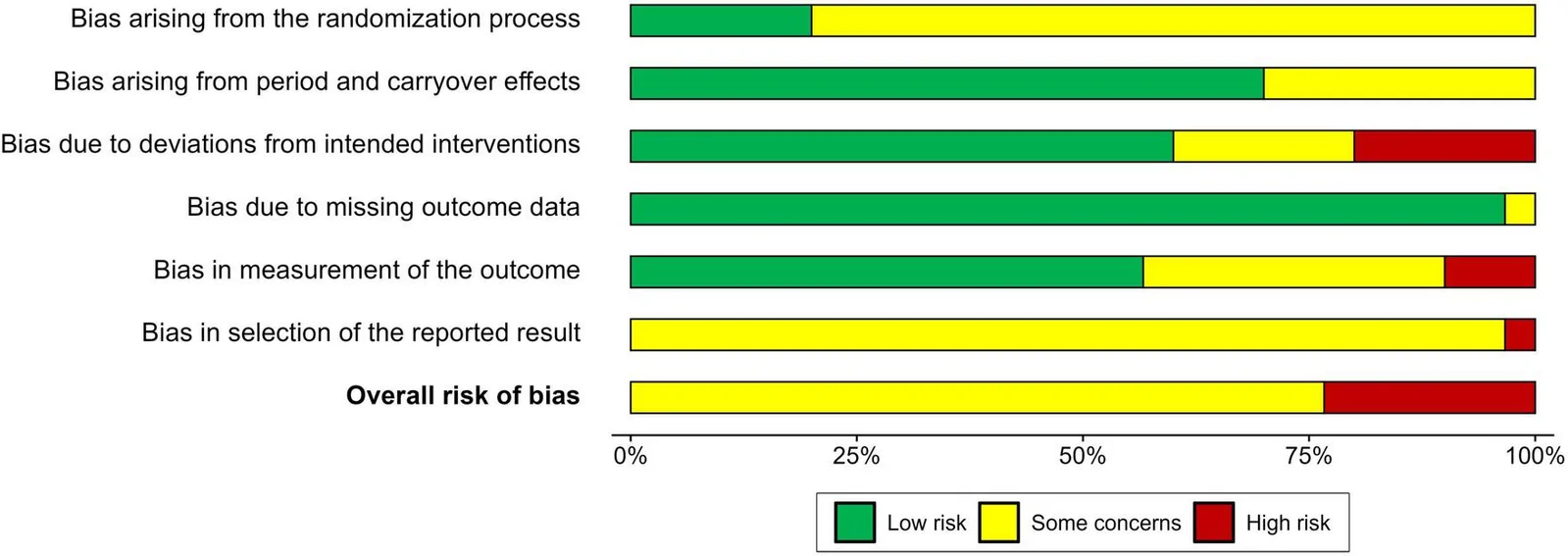
Summary risk-of-bias assessment for the subjective outcome of ratings of perceived exertion.

The summary risk-of-bias assessments based on the Cochrane RoB 2 tool are shown separately for objective performance outcomes and the subjective outcome of ratings of perceived exertion (RPE). Figure 3 presents the percentage distribution of risk-of-bias judgments for objective exercise performance outcomes, including time to exhaustion, work done during a fixed-time test, time to complete a fixed amount of work, and mean power output. Figure 4 presents the corresponding percentage distribution for the subjective outcome of RPE. The detailed risk-of-bias judgments for individual studies are provided in Supplementary file 1, with Supplementary Figure 4.1 showing objective exercise performance outcomes and Supplementary Figure 4.2 showing the subjective outcome of RPE.

The risk-of-bias assessments showed broadly similar patterns for objective performance outcomes and RPE. Most assessments were judged as having “some concerns” or “high risk” overall. The main sources of bias across both outcome categories were related to the randomization process, period and carryover effects, deviations from intended interventions, and selection of the reported result, whereas missing outcome data was generally less problematic. The main difference between the two outcome categories was observed in the measurement domain. Objective performance outcomes were usually judged as having lower risk of measurement bias because they were recorded using timing, distance, or power-output measures, whereas RPE was more susceptible to bias because it was self-reported and could be influenced by participants’ awareness of the assigned mouth-rinse condition. In particular, sensory characteristics such as menthol-related cooling sensation and caffeine-related bitterness were considered when judging potential deviations from intended interventions and measurement-related bias.

The certainty of evidence for each network comparison was further evaluated using the CINeMA framework. Overall, most comparisons across outcomes were rated as having low confidence, and no comparison was rated as high confidence. Only the CAF versus PLA comparison for time to exhaustion was rated as very low confidence. In general, most comparisons for time to complete a fixed amount of work, work done during a fixed-time test, mean power output, and RPE were rated as low confidence. The main reasons for downgrading were imprecision, within-study bias, and heterogeneity. These findings indicate that the certainty of the effect estimates and ranking probabilities remains limited and that the results should be interpreted with caution. The detailed CINeMA evidence confidence ratings are provided in Supplementary file 1:5.

### 3.3 Time to exhaustion

**FIGURE 5:**
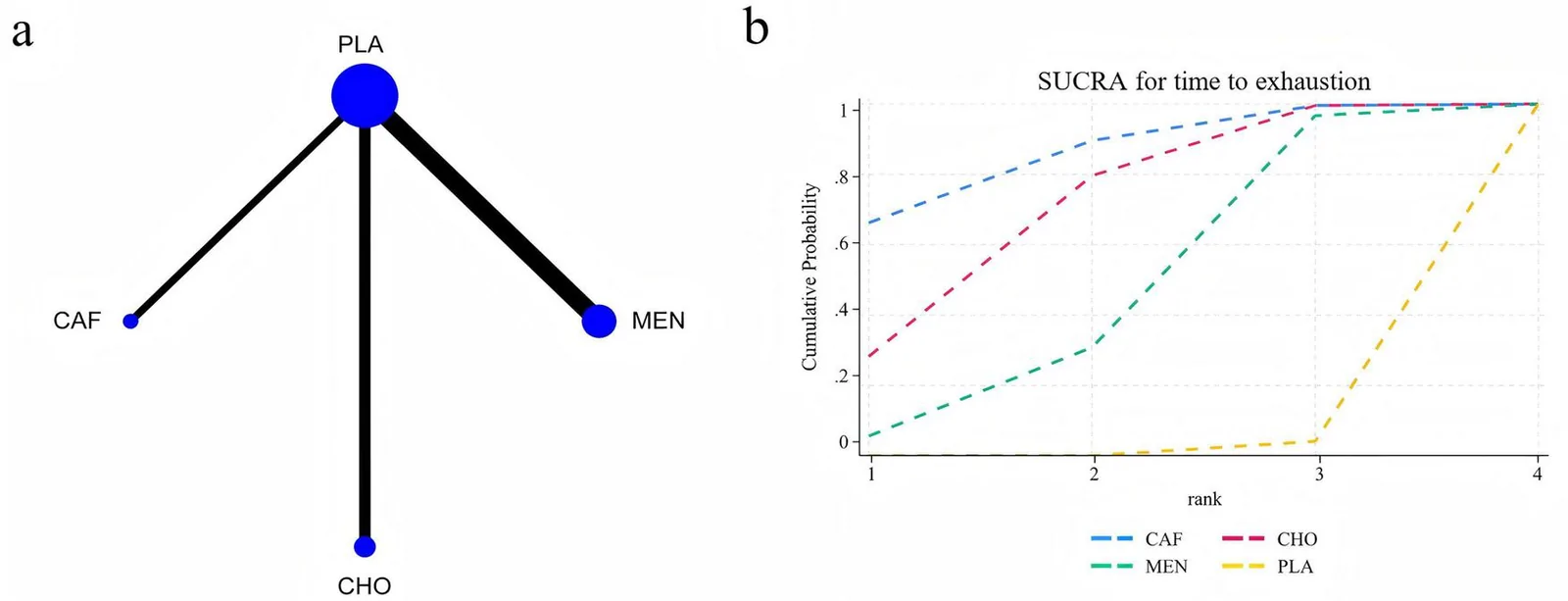
Network meta-analysis of time to exhaustion: network plot and SUCRA plot. **(a)** Network Plot. The size of the nodes is proportional to the sample size of each mouth-rinse intervention, and the thickness of the lines corresponds to the number of available studies. **(b)** The SUCRA plot, where the size of the area under the curve indicates the relative effectiveness of each intervention.

This review included 9 studies involving 111 participants that reported the outcome of time to exhaustion (46–54). The mouth-rinse ingredients included in the analysis were CAF, CHO, MEN, and PLA. Three studies compared CHO with PLA, two evaluated CAF versus PLA, and four compared MEN with PLA (Figure 5a).

Pairwise meta-analysis showed that, compared with PLA, both CHO (SMD = 0.43, 95% CI: 0.11, 0.75) and CAF (SMD = 0.57, 95% CI: 0.13, 1.01) significantly prolonged time to exhaustion, whereas MEN did not reach statistical significance (SMD = 0.27, 95% CI: −0.01, 0.56) (Supplementary file 2:6.1.1).

**FIGURE 6:**
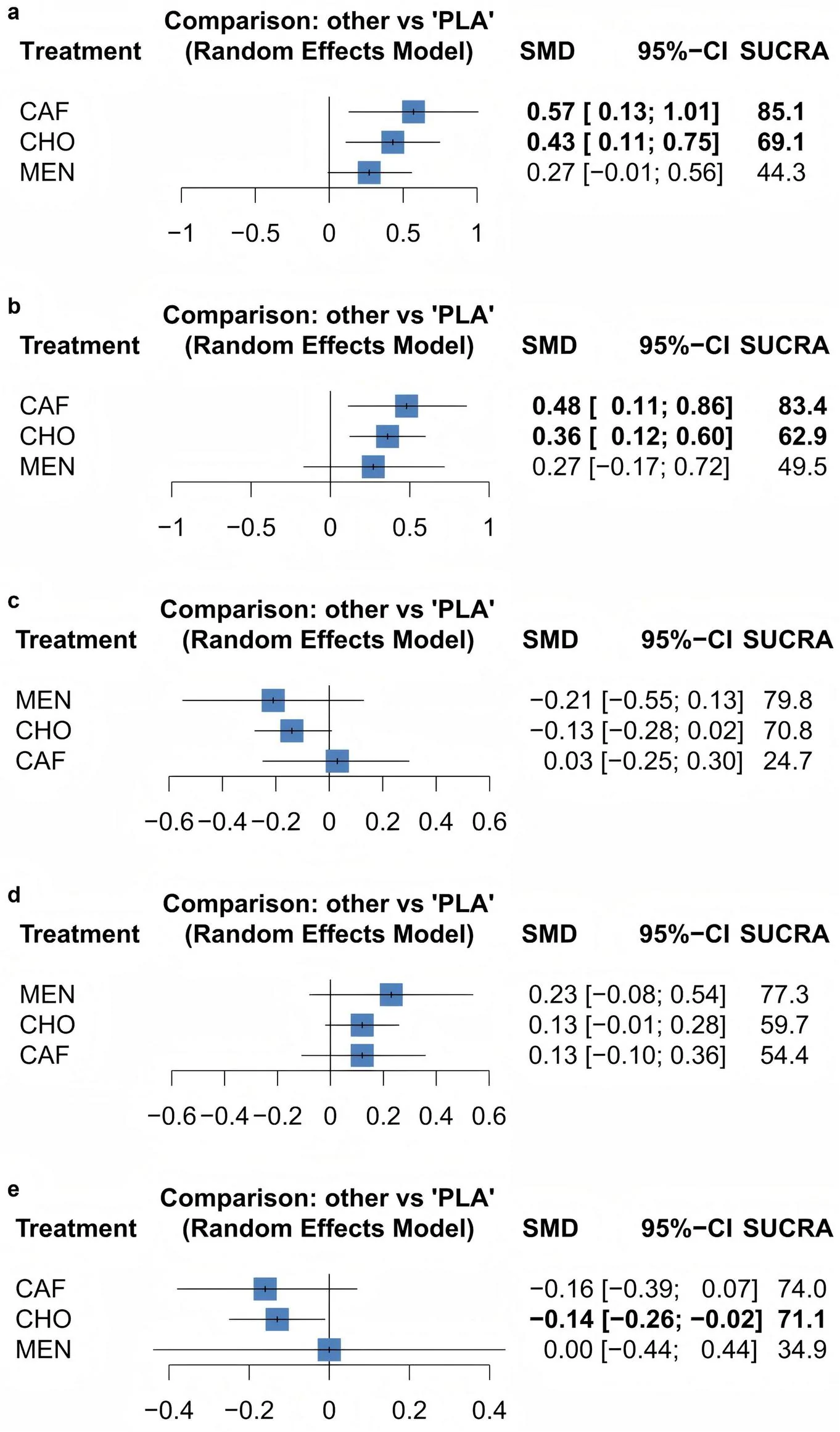
Forest plots of the network meta-analysis: **(a)** time to exhaustion; **(b)** work done during a fixed-time test; **(c)** time to complete a fixed amount of work; **(d)** mean power; **(e)** ratings of perceived exertion.

**FIGURE 7:**
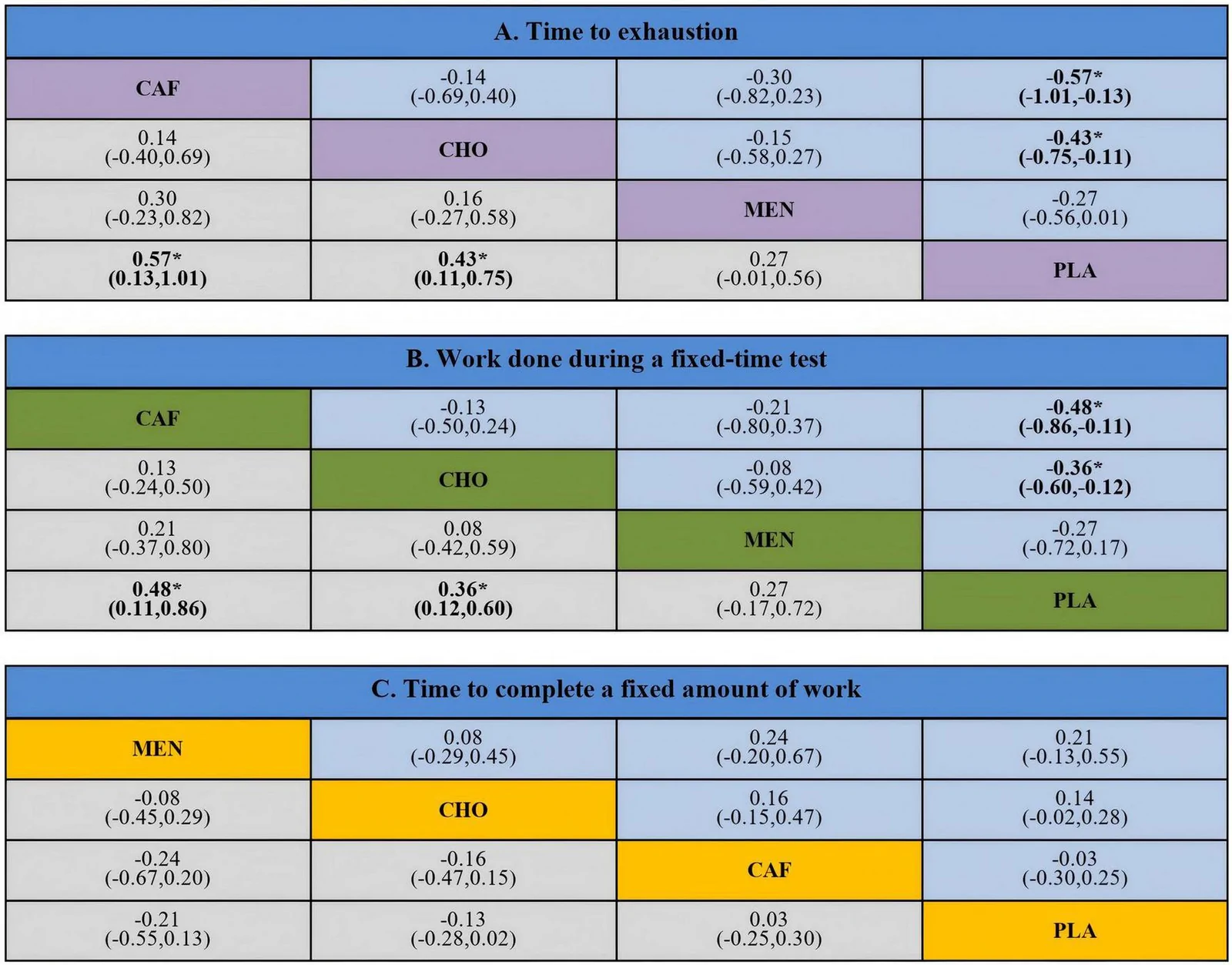
League-table results of the network meta-analysis. **(A)** Time to exhaustion. **(B)** Work done during a fixed-time test. **(C)** Time to complete a fixed amount of work. Values are SMDs with 95% CIs. Values shown in bold with an asterisk (*) indicate statistically significant comparisons.

Because the network contained no closed loops, inconsistency could not be formally assessed, and a consistency model was therefore used for the NMA. Compared with PLA, both CAF (SMD = 0.57, 95% CI: 0.13, 1.01) and CHO (SMD = 0.43, 95% CI: 0.11, 0.75) significantly prolonged time to exhaustion, whereas MEN did not show a statistically significant effect (SMD = 0.27, 95% CI: −0.01, 0.56) (Figure 6a). No statistically significant differences were observed among CAF, CHO, and MEN (Figure 7a). SUCRA rankings placed CAF first (85.1%), followed by CHO (69.1%), MEN (44.3%), and PLA (1.4%) (Figure 5b). Because no statistically significant differences were observed among CAF, CHO, and MEN, the SUCRA ranking should be interpreted as a probabilistic hierarchy rather than evidence of clear superiority.

### 3.4 Work done during a fixed-time test

**FIGURE 8:**
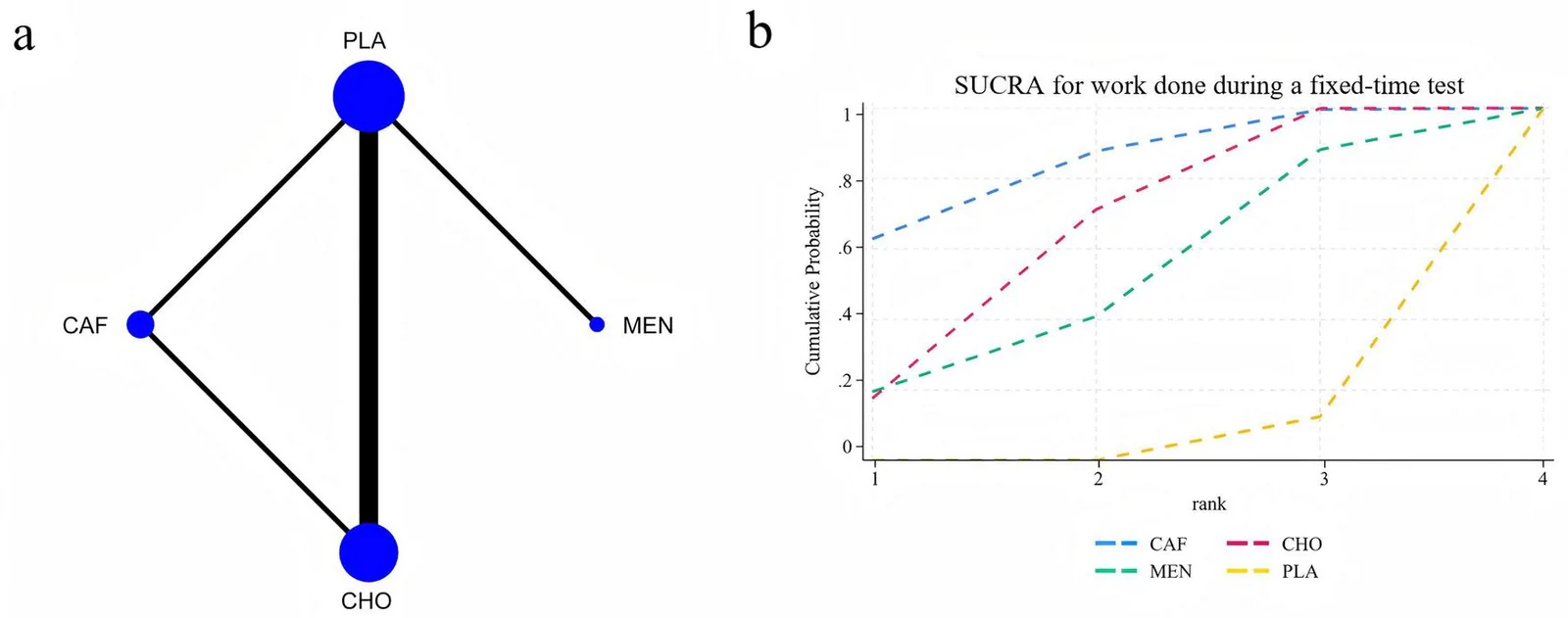
Network meta-analysis of work done during a fixed-time test: network plot and SUCRA plot. **(a)** Network plot. The size of the nodes is proportional to the sample size of each mouth-rinse intervention, and the thickness of the lines corresponds to the number of available studies. **(b)** The SUCRA Plot, where the size of the area under the curve indicates the relative effectiveness of each intervention.

A total of 9 studies involving 89 participants reported the outcome of work done during a fixed-time test (55–63). The mouth-rinse ingredients included in the analysis were CAF, CHO, MEN, and PLA. Five studies compared CHO with PLA, two evaluated CAF versus PLA, and two analyzed MEN versus PLA. Among these studies, two simultaneously evaluated CAF and CHO versus PLA (Figure 8a).

Pairwise meta-analysis showed that, compared with PLA, both CHO (SMD = 0.36, 95% CI: 0.11, 0.60) and CAF (SMD = 0.56, 95% CI: 0.14, 0.98) significantly increased work done during a fixed-time test, whereas MEN did not show a statistically significant effect (SMD = 0.27, 95% CI: −0.17, 0.72) (Supplementary file 2:6.2.1).

The global inconsistency test showed no evidence of significant inconsistency (*p* = 0.462). Node-splitting analyses also indicated no significant local inconsistency. Therefore, a consistency model was used for the NMA. Compared with PLA, both CAF (SMD = 0.48, 95% CI: 0.11, 0.86) and CHO (SMD = 0.36, 95% CI: 0.12, 0.60) significantly increased work done during a fixed-time test, whereas MEN did not show a statistically significant effect (SMD = 0.27, 95% CI: −0.17, 0.72) (Figure 6b). No statistically significant differences were observed among CAF, CHO, and MEN (Figure 7b). SUCRA rankings placed CAF first (83.4%), followed by CHO (62.9%), MEN (49.5%), and PLA (4.2%) (Figure 8b). Because no statistically significant differences were observed among CAF, CHO, and MEN, the SUCRA ranking should be interpreted as a probabilistic hierarchy rather than evidence of clear superiority.

### 3.5 Time to complete a fixed amount of work

**FIGURE 9:**
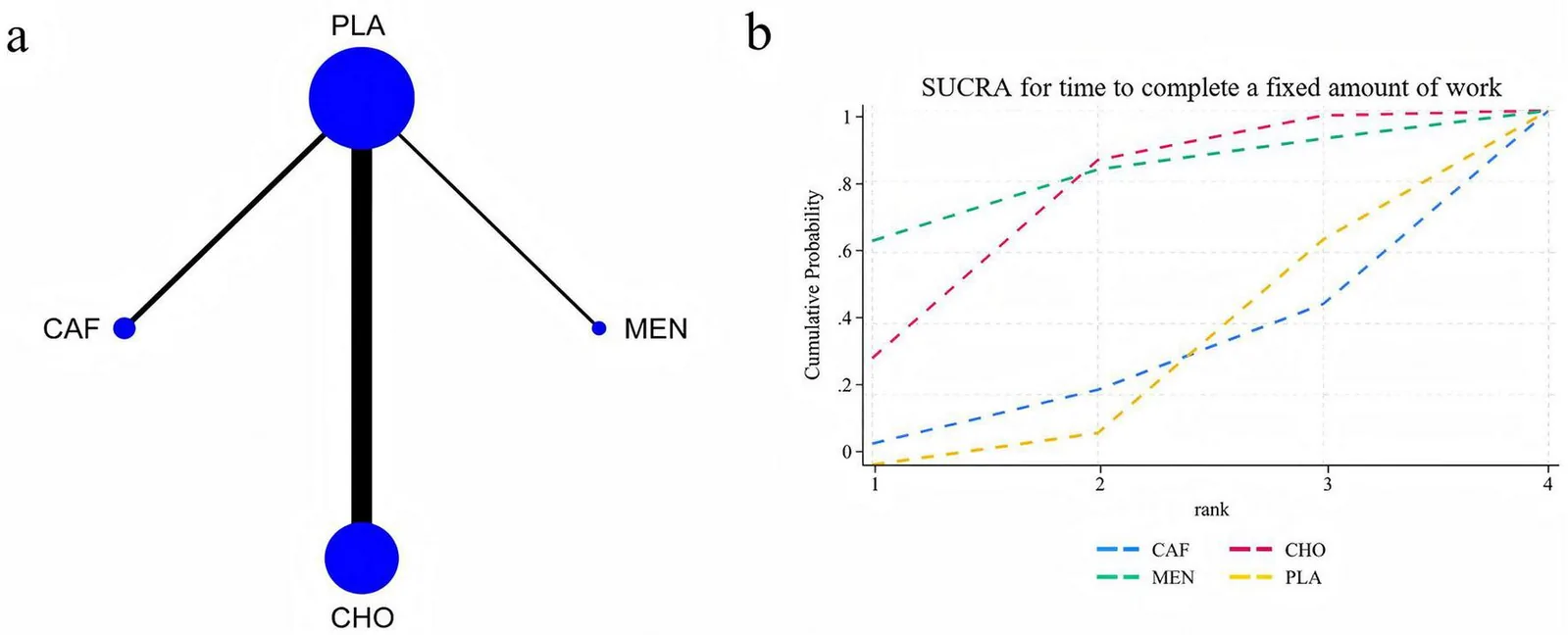
Network meta-analysis of time to complete a fixed amount of work: network plot and SUCRA plot. **(a)** Network Plot. The size of the nodes is proportional to the sample size of each mouth-rinse intervention, and the thickness of the lines corresponds to the number of available studies. **(b)** The SUCRA Plot, where the size of the area under the curve indicates the relative effectiveness of each intervention.

For the outcome of time to complete a fixed amount of work, 24 studies involving 260 participants were included (10, 64–86). The mouth-rinse ingredients included in the analysis were CAF, CHO, MEN, and PLA. Sixteen studies evaluated CHO versus PLA, five compared CAF with PLA, and three evaluated MEN versus PLA (Figure 9a).

Pairwise meta-analysis showed that, compared with PLA, CHO (SMD = −0.13, 95% CI: −0.28, 0.02), CAF (SMD = 0.03, 95% CI: −0.25, 0.30), and MEN (SMD = −0.21, 95% CI: −0.55, 0.13) did not show statistically significant effects on the time required to complete a fixed amount of work (Supplementary file 2:6.3.1).

Because the network contained no closed loops, inconsistency could not be formally assessed, and node-splitting analysis was not feasible. Therefore, a consistency model was used for the NMA. The NMA showed no statistically significant differences between CAF, CHO, or MEN and PLA (Figure 6c), nor among CAF, CHO, and MEN (Figure 7c). SUCRA rankings placed MEN first (79.8%), followed by CHO (70.8%), while CAF and PLA had the same SUCRA value (both 24.7%) (Figure 9b). Because no statistically significant differences were observed among CAF, CHO, and MEN, the SUCRA ranking should be interpreted as a probabilistic hierarchy rather than evidence of clear superiority.

Exploratory meta-regression was performed for the CHO versus PLA comparison, with participants’ age (*P* = 0.614), sample size (*P* = 0.687), and rinse duration (*P* = 0.293) as covariates. The results showed that none of these covariates was significantly associated with the intervention effect on time to complete a fixed amount of work (Supplementary file 2:6.3.1).

### 3.6 Mean power

**FIGURE 10:**
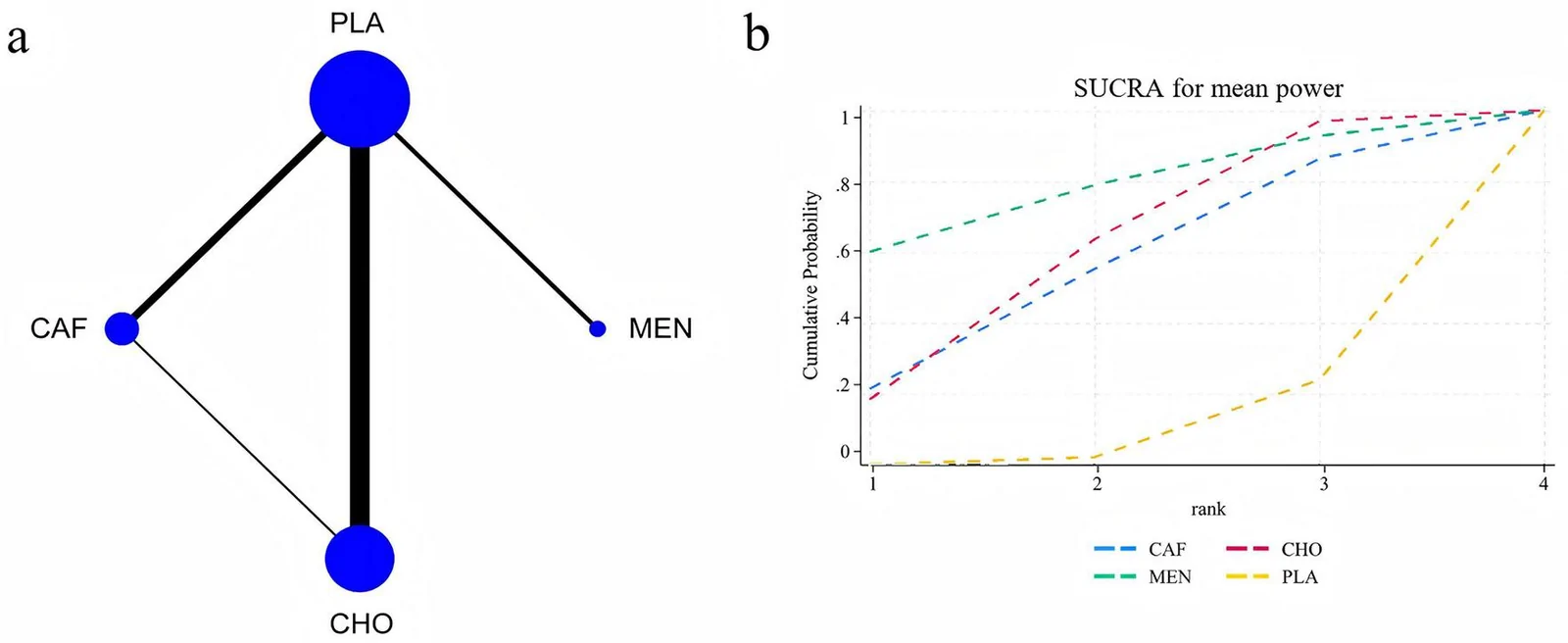
Network meta-analysis of mean power: network plot and SUCRA plot. **(a)** Network plot. The size of the nodes is proportional to the sample size of each mouth-rinse intervention, and the thickness of the lines corresponds to the number of available studies. **(b)** The SUCRA Plot, where the size of the area under the curve indicates the relative effectiveness of each intervention.

For the outcome of mean power, 24 studies involving 268 participants were included (10, 46, 47, 55–58, 64–73, 87–93). The mouth-rinse ingredients included in the analysis were CAF, CHO, MEN, and PLA. Fourteen studies compared CHO with PLA, seven evaluated CAF versus PLA, and three compared MEN with PLA. Among these studies, two simultaneously evaluated CAF and CHO versus PLA (Figure 10a).

Pairwise meta-analysis showed that, compared with PLA, CHO (SMD = 0.14, 95% CI: −0.01, 0.29), CAF (SMD = 0.14, 95% CI: −0.30, 0.57), and MEN (SMD = 0.23, 95% CI: −0.08, 0.54) did not show statistically significant effects on mean power (Supplementary file 2:6.4.1).

**FIGURE 11:**
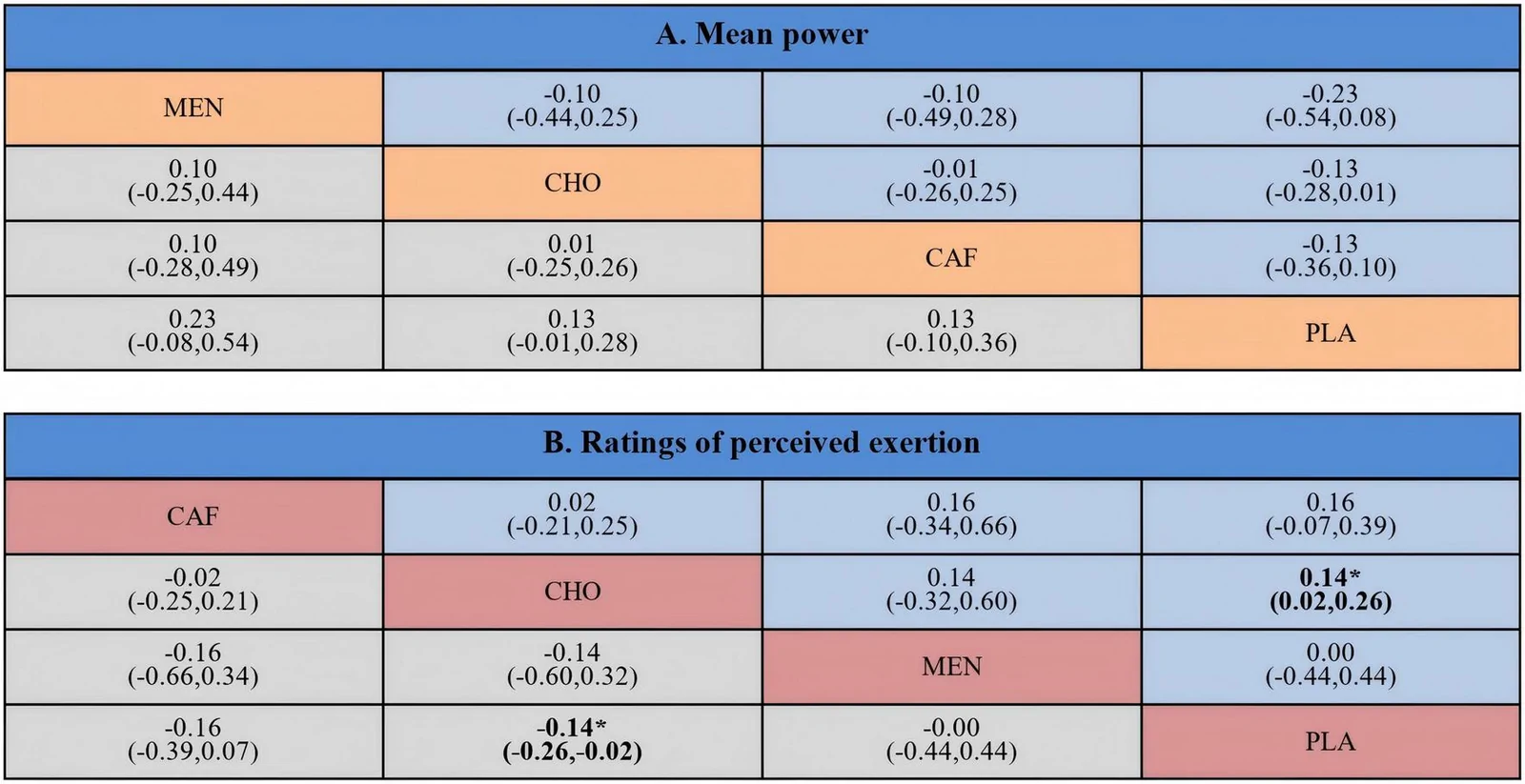
League-table results of the network meta-analysis. **(A)** Mean power output. **(B)** Ratings of perceived exertion. Values are SMDs with 95% CIs. Values shown in bold with an asterisk (*) indicate statistically significant comparisons.

The global inconsistency test did not indicate significant inconsistency (*p* = 0.388). Node-splitting analyses likewise indicated no significant local inconsistency. Therefore, a consistency model was used for the NMA. The NMA showed no statistically significant differences between CAF, CHO, or MEN and PLA, or among CAF, CHO, and MEN (Figures 6d, 11a). SUCRA rankings placed MEN first (77.3%), followed by CHO (59.7%), CAF (54.4%), and PLA (8.7%) (Figure 10b). Because no statistically significant differences were observed among CAF, CHO, and MEN, the SUCRA ranking should be interpreted as a probabilistic hierarchy rather than evidence of clear superiority.

Exploratory meta-regression was performed for the CHO versus PLA comparison, with participants’ age (*P* = 0.810), sample size (*P* = 0.520), and rinse duration (*P* = 0.569) as covariates. The results showed that none of these covariates was significantly associated with the intervention effect on mean power (Supplementary file 2: 6.4.1).

### 3.7 Ratings of perceived exertion

**FIGURE 12:**
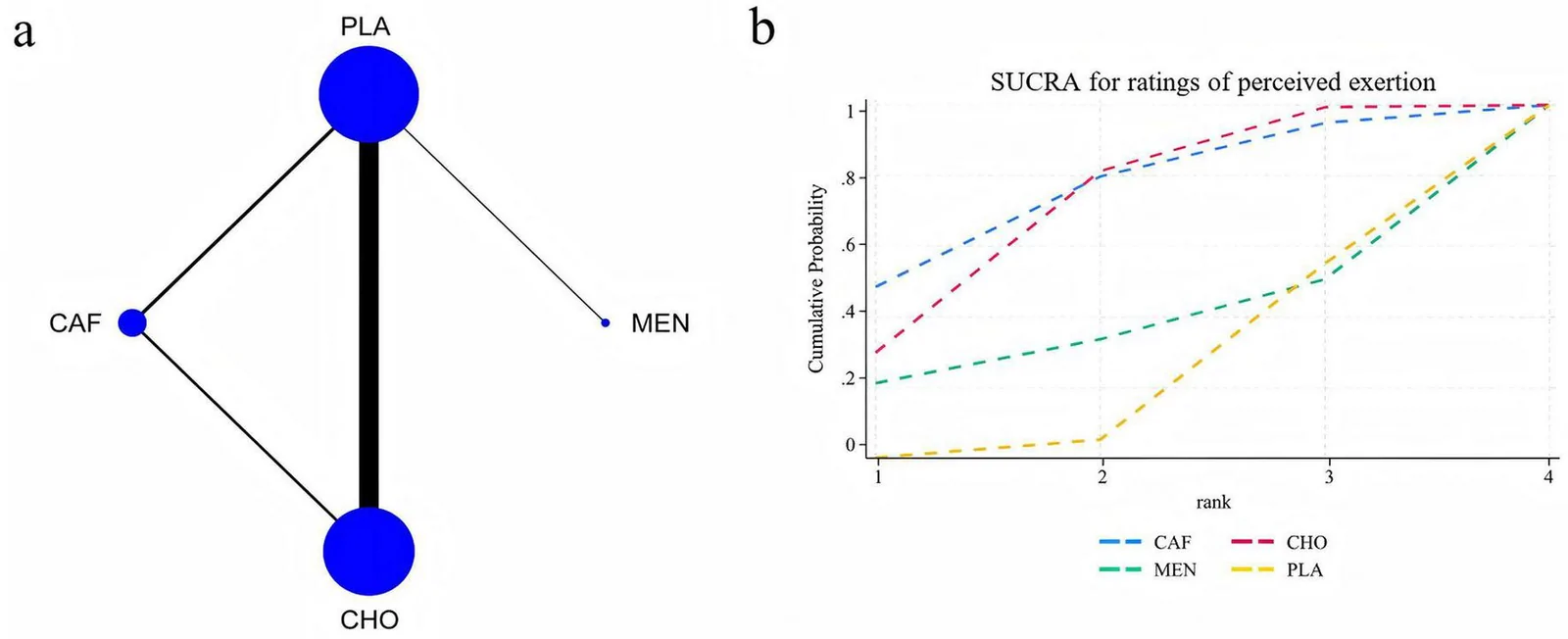
Network meta-analysis of ratings of perceived exertion: network plot and SUCRA plot. **(a)** Network plot. The size of the nodes is proportional to the sample size of each mouth-rinse intervention, and the thickness of the lines corresponds to the number of available studies. **(b)** The SUCRA Plot, where the size of the area under the curve indicates the relative effectiveness of each intervention.

A total of 30 studies with 337 participants were included in the outcome of RPE (10, 48, 49, 55–60, 64–69, 74–79, 93–101). The mouth-rinse ingredients included in the analysis were CAF, CHO, MEN, and PLA. The relevant direct comparisons involved CAF versus PLA, CHO versus PLA, and MEN versus PLA, and some studies included multiple intervention arms, thereby forming a network structure including CAF, CHO, MEN, and PLA (Figure 12a).

Pairwise meta-analysis showed that, compared with PLA, CHO significantly reduced RPE (SMD = −0.13, 95% CI: −0.25, −0.02), whereas CAF (SMD = −0.16, 95% CI: −0.51, 0.20) and MEN (SMD = 0.00, 95% CI: −0.42, 0.42) did not show statistically significant effects (Supplementary file 2:6.5.1).

The overall inconsistency test showed no evidence of significant inconsistency (*P* = 0.575). Node-splitting analyses also indicated no significant local inconsistency. Therefore, a consistency model was used for the NMA. Compared with PLA, CHO significantly reduced RPE (SMD = −0.14, 95% CI: −0.26, −0.02), whereas CAF (SMD = −0.16, 95% CI: −0.39, 0.07) and MEN (SMD = 0.00, 95% CI: −0.44, 0.44) did not show statistically significant effects (Figure 6e). No statistically significant differences were observed among CAF, CHO, and MEN (Figure 11b). SUCRA rankings placed CAF first (74.0%), followed by CHO (71.1%), MEN (34.9%), and PLA (20.0%) (Figure 12b). Because no statistically significant differences were observed among CAF, CHO, and MEN, the SUCRA ranking should be interpreted as a probabilistic hierarchy rather than evidence of clear superiority.

Exploratory meta-regression was performed for the CHO versus PLA comparison, with participants’ age (*P* = 0.928), sample size (*P* = 0.798), and rinse duration (*P* = 0.090) as covariates. The results showed that none of these covariates was significantly associated with the intervention effect on RPE (Supplementary file 2:6.5.1).

### 3.8 Sensitivity analysis

Sensitivity analyses using within-participant correlation coefficients of *r* = 0.30 and *r* = 0.70 produced effect estimates that were similar in magnitude and direction to those of the primary analysis based on *r* = 0.50. The principal effect of changing the assumed correlation coefficient was therefore on the width of the confidence intervals and, consequently, on the statistical significance of several comparisons close to the null value. Under the *r* = 0.30 assumption, the pairwise effect of CHO versus PLA on ratings of perceived exertion was no longer statistically significant. Under the *r* = 0.70 assumption, MEN versus PLA for time to exhaustion and CHO versus PLA for time to complete a fixed amount of work became statistically significant in both the pairwise and network meta-analyses; CHO versus PLA for mean power was significant only in the pairwise meta-analysis. The highest-ranked intervention for each outcome remained unchanged, and no statistically significant differences were observed among CAF, CHO, and MEN under either alternative assumption. These findings indicate that the overall effect patterns and treatment rankings were stable, although the statistical significance of several borderline comparisons depended on the assumed within-participant correlation coefficient (Supplementary file 2:7).

### 3.9 Publication bias

This study evaluated publication bias via funnel plots and Egger’s regression test. Egger’s regression test did not detect statistically significant small-study effects for time to exhaustion (*P* = 0.4741), work done during a fixed-time test (*P* = 0.8281), time to complete a fixed amount of work (*P* = 0.7950), mean power (*P* = 0.4488), or RPE (*P* = 0.9161). These results suggest no statistical evidence of significant publication bias based on Egger’s tests. However, because several comparisons included a limited number of studies and small sample sizes, the results of publication-bias assessment should be interpreted cautiously. Detailed Egger’s regression results are provided in Supplementary file 2:Sections 6.1.7–6.5.7, and the comparison-adjusted funnel plots are shown in Supplementary file 2:Sections 6.1.8–6.5.8.

## 4 Discussion

This systematic review and network meta-analysis is the first to systematically evaluate the effects of CHO, CAF, and MEN mouth rinses on different types of exercise performance and subjective perception (RPE). Results showed that the relative advantages of mouth rinses containing different ingredients varied across exercise performance and perceived exertion outcomes. Among the objective performance outcomes, CAF mouth rinse ranked first for work done during a fixed-time test and time to exhaustion, whereas MEN mouth rinse ranked first for time to complete a fixed amount of work and mean power. For the subjective outcome of RPE, CAF mouth rinse ranked first, closely followed by CHO mouth rinse, with similar SUCRA values. Notably, although the SUCRA rankings suggested outcome specific differences in the relative rankings of mouth-rinse ingredients, the league tables and forest plots indicated that most network comparisons were not statistically significant. Thus, the current evidence supported probabilistic and trend-based conclusions rather than definitive judgments of superiority. Overall, the findings of this study demonstrated that mouth rinses with different ingredients may exert distinct effects on varying types of exercise performance. However, the existing evidence is insufficient to determine a single optimal intervention. In addition, among the comparisons eligible for meta-regression, no statistically significant associations were identified between mean participant age, study sample size, rinse duration, and intervention effects. To provide a concise overview of the main network meta-analysis findings across outcomes, the significant NMA results versus PLA and SUCRA rankings are summarized in Table 2.

**TABLE 2:** Summary of network meta-analysis results across outcomes.

Outcome	Studies/participants	Significant NMA finding vs. PLA	SUCRA ranking
Time to exhaustion	9/111	CAF vs. PLA: SMD = 0.57, 95% CI: 0.13–1.01 CHO vs. PLA: SMD = 0.43, 95% CI: 0.11–0.75	CAF 85.1% > CHO 69.1% > MEN 44.3% > PLA 1.4%
Work done during a fixed-time test	9/89	CAF vs. PLA: SMD = 0.48, 95% CI: 0.11–0.86 CHO vs. PLA: SMD = 0.36, 95% CI: 0.12–0.60	CAF 83.4% > CHO 62.9% > MEN 49.5% > PLA 4.2%
Time to complete a fixed amount of work	24/260	No significant difference vs. PLA	MEN 79.8% > CHO 70.8% > CAF 24.7% > PLA 24.7%*
Mean power	24/268	No significant difference vs. PLA	MEN 77.3% > CHO 59.7% > CAF 54.4% > PLA 8.7%
Ratings of perceived exertion	30/337	CHO vs. PLA: SMD = −0.14, 95% CI: −0.26 to −0.02	CAF 74.0% > CHO 71.1% > MEN 34.9% > PLA 20.0%

*For time to complete a fixed amount of work, CAF and PLA both round to 24.7% when SUCRA values are presented to one decimal place; however, when reported to two decimal places, the SUCRA values for CAF was slightly higher than that for PLA. Therefore, CAF was ranked above PLA.

### 4.1 Summary of the main findings

The relative advantages of mouth rinses containing different ingredients were inconsistent across exercise performance outcomes. This suggested that these differences may not be attributable solely to the names of the outcomes themselves but may also be related to differences in the task demands associated with those outcomes. Therefore, the included studies must be systematically categorized based on the testing framework used for the exercise tasks. The exercise tasks used in the existing literature can be broadly divided into constant-load protocols and self-paced protocols (102). Constant-load protocols generally require participants to exercise continuously at a predetermined intensity until they can no longer sustain that load. Their typical outcome is time to exhaustion (TTE), which mainly reflects the ability to sustain exercise at a given level of physiological stress (103). By contrast, self-paced protocols allow participants to actively regulate output intensity and pacing strategy during the task based on their own condition. Typical performance outcomes include work done during a fixed-time test and time required to complete a fixed amount of work. These indicators may partially reflect an individual’s ability to dynamically regulate exercise output (104, 105). Although mean power is not an independent task type, it is often used as a performance indicator in self-paced tasks and can reflect overall output level (106). Accordingly, the mechanisms by which different mouth-rinse strategies function may vary under these two regulatory contexts. In constant-load protocols, their effects are likely to be expressed as a delay in fatigue progression or as support for sustaining high-load exercise, whereas in self-paced protocols, their potential effects may involve perceived effort, pacing adjustment, and output distribution (107).

For the constant-load outcome of time to exhaustion, CAF mouth rinse had the highest SUCRA value (85.1%), followed by CHO (69.1%), whereas MEN ranked lower (44.3%) and PLA ranked lowest (1.4%). This ranking pattern suggests that, in task settings primarily constrained by the upper limit of physiological tolerance, CAF mouth rinse may have the highest probability of producing an ergogenic benefit. However, when the league table and forest plot were considered together, both CAF and CHO showed statistically significant benefits compared with PLA, whereas MEN did not. No statistically significant differences were observed among CAF, CHO, and MEN. Therefore, the TTE findings support beneficial effects of both CAF and CHO relative to PLA, while the higher SUCRA ranking of CAF should be interpreted as a probabilistic hierarchy rather than evidence of superiority over CHO or MEN. In constant-load protocols, external intensity is predetermined, and task failure is primarily influenced by the ongoing accumulation of fatigue perception. Participants have almost no opportunity to mitigate fatigue by adjusting pace. Thus, improvements in TTE are likely to be related to the modulation of fatigue perception, a slower rise in perceived effort, and improved preservation of central drive (108). Against this background, CAF’s leading rank may have a plausible mechanistic basis. As a bitter substance, CAF may activate oral bitter-related receptors and act through ascending neural pathways on central brain regions related to motivation and cognitive regulation, thereby helping delay the accumulation of fatigue perception and maintain central drive in constant-load tasks (109). Previous studies have suggested that oral CAF rinsing alone can activate prefrontal regions such as the orbitofrontal cortex, which may serve as a crucial mechanism for regulating fatigue perception and sustaining central drive (110). Notably, this process is independent of gastrointestinal absorption and peripheral metabolic fuel supply, allowing CAF to enter central regulation rapidly after oral exposure. This interpretation is supported, although not uniformly, by experimental studies. Melo et al. (49) observed in a constant-load cycling-to-task-failure study at moderate intensity that CAF mouth rinse significantly increased TTE by approximately 17% and was accompanied by reduced electromyographic activity in the vastus lateralis and rectus femoris. By contrast, Nabuco et al. (52) observed a numerically longer TTE under the CAF condition, but the difference did not reach statistical significance. These findings indicate that, although statistical significance varied between individual studies, both showed a favorable direction of effect. In the present review, the pooled estimate indicated a statistically significant benefit of CAF compared with PLA.

Although CHO and MEN also ranked above PLA for TTE, the reasons for their intermediate positions may differ from those of CAF. Previous studies have shown that CHO mouth rinse can prolong TTE during constant-load exercise, and such improvements often occur without obvious changes in peripheral indicators such as blood glucose, lactate, and heart rate (50, 51). This suggests that its mechanism may be more closely related to the activation of reward circuits through oral carbohydrate sensing and the modulation of perceived effort than to direct peripheral metabolic support (4, 7). In the present study, CHO showed a statistically significant benefit compared with PLA, suggesting that it may also contribute to the maintenance of fatigue tolerance during constant-load exercise. The main mechanism of MEN is considered to involve improvements in thermal sensation and subjective comfort through oral cold receptors (111). However, because exercise intensity is predetermined in constant-load tasks, participants have limited opportunity to use these perceptual changes to adjust their pacing (112). As a result, the perceptual benefits of MEN may not be fully translated into prolonged exercise tolerance, which may partly explain why its effect on TTE did not reach statistical significance. Overall, CAF and CHO may enhance fatigue tolerance through different oral sensory and central regulatory pathways, whereas the potential effects of MEN may depend more strongly on the exercise environment and the opportunity to adjust pacing. However, no statistically significant differences were observed among CAF, CHO, and MEN, and their relative rankings should therefore be interpreted as probabilistic trends rather than clear differences in efficacy.

Unlike constant-load protocols, self-paced protocols allow participants to actively regulate output under a given duration or task requirement. Therefore, the advantages of mouth rinses containing different ingredients are no longer determined solely by fatigue tolerance itself, but they may also be influenced by pacing strategy, output distribution, and the way in which the task is completed. In the present NMA, the outcomes derived from self-paced protocols were mainly represented by work done during a fixed-time test and time to complete a fixed amount of work. For work done during a fixed-time test, the ranking pattern in the present study showed some directional consistency with TTE, with CAF again showing the most favorable ranking trend. Its SUCRA value (83.4%) was higher than those of CHO (62.9%) and MEN (49.5%), whereas PLA ranked last (4.2%). Both CAF and CHO showed statistically significant benefits compared with PLA, whereas MEN did not. Fixed-time tasks require participants to maintain a relatively high level of external output as fatigue gradually accumulates over a prescribed duration (102). The central arousal and motivational effects associated with CAF mouth rinsing may help participants maintain exercise drive during self-paced exercise. In a 30-min self-paced cycling trial, Bottoms et al. (56) reported that CAF mouth rinse improved total cycling distance, cadence, and power output compared with PLA, without marked increases in heart rate or RPE. Exercise performance under the CHO mouth-rinse condition was also better than under PLA, although CAF showed more favorable results than CHO for several output-related measures. Other studies have indicated that the effects of CHO mouth rinse may vary according to exercise mode and rinse protocol. Whitham and McKinney (61) observed no significant benefit during a 45-min running time trial, whereas Sinclair et al. (55) reported that longer rinse durations may provide benefits during 30-minute self-paced cycling. These findings suggest that both CAF and CHO mouth rinses may support performance during fixed-duration exercise, although their effects may vary across experimental protocols. MEN ranked below CAF and CHO and did not show a statistically significant benefit compared with PLA. Its potential effects may depend more strongly on environmental conditions because improvements in thermal sensation and thermal comfort may be more relevant when heat discomfort is an important limiting factor (21, 46). No statistically significant differences were observed among CAF, CHO, and MEN. This finding indicates that their relative rankings should be interpreted as probabilistic patterns rather than clear differences in efficacy.

Unlike work done during a fixed-time test, which emphasizes the ability to maintain high output over a prescribed duration, time to complete a fixed amount of work places greater emphasis on pacing distribution and completion efficiency under a fixed total-work requirement. Notably, for this outcome, the ranking pattern in the present study differed markedly from those observed for TTE and work done during a fixed-time test. CAF mouth rinse (24.7%) ranked lowest among the three active interventions and was close to PLA (24.7%), whereas MEN (79.8%) and CHO (70.8%) ranked near the top. This shift suggested that the relative advantages of different ingredients were not fixed but likely to depend on the degree of fit between their mode of action and the specific demands of the task. In tasks based on time to complete a fixed amount of work, participants must continuously integrate the amount of work remaining, their current fatigue level, and their subjective tolerability to achieve an appropriate pacing distribution (113). In this context, MEN’s leading rank may be mechanistically related to its effects on thermal sensation and subjective comfort. By activating oral cold receptors (TRPM8), MEN improves thermal sensation and subjective comfort. Its effect is not to enhance maximal output capacity but rather to reduce perceptual burden during the task, thereby expanding the subjective comfort zone within which participants are willing to sustain output and making pacing adjustment stable and power distribution across the task appropriate. This perceptual regulatory effect may be compatible with the pacing precision and completion efficiency required in this type of task. Whether MEN’s support for pacing regulation under temperate conditions is fully independent of heat load still requires further study. Gavel et al. (18) suggested in a meta-analysis that its effects are pronounced in hot environments, whereas not all fixed-work studies included in the present analysis were conducted under heat stress. Thus, the conditions under which MEN is likely to produce ergogenic benefits in temperate pacing tasks remain to be clarified. CHO ranked behind MEN. Its reward-related effects may help preserve a relatively stable output rhythm, but its direct influence on fine-grained perceptual regulation may be less pronounced than that of MEN. Brietzke et al. (27) reported in a meta-analysis that CHO mouth rinse did not significantly improve the time to complete a fixed amount of work, although it improved mean power to some extent, suggesting that its effects may be expressed more in maintaining output quality than in systematically shortening completion time. By contrast, CAF’s low rank for this outcome suggests that its mechanism may not be fully aligned with the core demands of this type of task. The heightened central arousal state induced by bitter receptor activation may be advantageous in tasks requiring sustained high output. However, in timed tasks that demand precise pacing, this arousal effect may interfere with participants’ accurate appraisal of their current effort and remaining reserves, leading to excessive output early in the task, deviation from the optimal pacing range, and subsequent slowing as fatigue accumulates prematurely. This is not favorable for shortening completion time. Doering et al. (84) observed no significant effect of CAF mouth rinse in a 1-h cycling test at 75% Wpeak, and Boat et al. (83) likewise found no benefit in a 10-km cycling time trial. Together, these findings suggest that the effects of CAF mouth rinse remain uncertain in timed tasks where completion time is the primary outcome. Overall, for the outcome of time to complete a fixed amount of work, fine-grained perceptual regulation and compatibility with pacing strategy may be more important than the intensity of central arousal.

In addition to time to exhaustion, work done during a fixed-time test, and time to complete a fixed amount of work, mean power is another commonly reported performance indicator that reflects average output throughout exercise (114, 115). The present study showed that the SUCRA ranking for mean power was highly similar to that for time to complete a fixed amount of work, with MEN again ranking first (77.3%), CHO (59.7%), and CAF (54.4%) showing similar rankings, and PLA remaining last (8.7%). The cross-outcome consistency between these two indicators has an inherent measurement rationale. In fixed-work tasks, a shorter completion time means a higher average output per unit of time. Thus, the ranking pattern for mean power is essentially a mirror reflection of the ranking pattern for completion time. The two outcomes indicate that interventions aimed at reducing perceptual burden may be superior to central-arousal interventions in preserving output efficiency during fine-paced tasks. From the perspective of mean power, MEN’s higher ranking was also reflected in the mean-power outcome. Given that MEN may reduce perceptual stress during the task by improving thermal sensation and subjective comfort, participants may be able to maintain a higher sustainable output throughout the task rather than overextending early and then experiencing a sharp drop in power later, which would lower the overall average. This potential mechanism may partly account for the relatively high SUCRA ranking of MEN for mean power (18, 46, 63). Notably, the difference between CAF and CHO for mean power was small (54.4% vs. 59.7%), whereas their ranking difference for time to complete a fixed amount of work was greater (24.7% vs. 70.8%). This discrepancy suggests that CAF’s central arousal effect may partially support single-bout output quality. However, it may interfere with pacing strategy, so this output quality is not effectively translated into a shortened completion time. In fixed-work tasks, increased overall output does not necessarily mean improved completion time; pacing control is equally important.

Unlike the exercise performance outcomes discussed above, RPE reflects an integrated result of how individuals combine effort level, fatigue signals, emotional state, and sensory input during exercise (116, 117). In the present study, CAF ranked first for RPE (74.0%), closely followed by CHO (71.1%), whereas MEN (34.9%) and PLA (20.0%) ranked relatively low. However, only CHO showed a statistically significant reduction in RPE compared with PLA. Given the small difference between the SUCRA values of CAF and CHO, their ranking probabilities were broadly similar, and the slightly higher ranking of CAF should not be interpreted as evidence of a stronger effect. CAF mouth rinse may influence perceived exertion through signaling related to bitter receptors and central arousal, but findings from individual studies have been inconsistent. Doering et al. (84) observed no significant effect of CAF on RPE, performance, or physiological responses during endurance cycling, and Figueiredo et al. (78) likewise found no reduction in RPE during a 10-km run. These findings suggest that the influence of CAF on perceived exertion may depend on the exercise task and intervention protocol, rather than showing a consistent reduction across exercise contexts. CHO ranked closely behind CAF and was the only ingredient that significantly reduced RPE compared with PLA. This effect may be related to the unique nature of oral CHO signaling. Once the oral cavity detects CHO-related cues, individuals may subjectively perceive that energy is becoming available. The sweetness of CHO mouth rinse may also contribute to this response by enhancing pleasure and expectancy, although previous studies have suggested that oral CHO recognition may not depend entirely on sweet-taste pathways (75). Although CHO is not ingested or metabolized, oral stimulation may lead the brain to form a more favorable judgment of the current energy state, thereby slowing the accumulation of subjective fatigue and preventing RPE from increasing in parallel with exercise output. Carter et al. (64) found in a 1-h cycling time trial that CHO mouth rinse improved completion time and increased mean power without a significant increase in RPE. Devenney et al. (65) observed a similar pattern in the fed state, in which CHO improved performance while changes in RPE remained limited. Under mental fatigue, Brietzke et al. (22) further found that CHO did not significantly alter RPE but attenuated the decline in performance. Although these individual studies did not consistently demonstrate a direct reduction in RPE, they suggest that CHO may help maintain or improve performance without a proportional increase in perceived exertion. Compared with CAF and CHO, MEN ranked relatively low for RPE. Existing studies suggest that MEN mouth rinse primarily influences thermal sensation and thermal comfort, and its influence on RPE may depend on whether heat stress is an important limiting factor. Flood et al. (46) found that MEN mouth rinse increased power output and prolonged exercise duration while participants maintained the same prescribed RPE, together with a reduction in thermal sensation. This finding indicates that MEN may allow greater exercise output at a similar perceived effort rather than directly reducing RPE. Gibson et al. (118) similarly reported improved thermal comfort but no significant effect on RPE or intermittent sprint performance. Overall, CAF and CHO showed similar ranking probabilities for RPE, but only CHO demonstrated a statistically significant benefit compared with PLA. No statistically significant differences were observed among CAF, CHO, and MEN, indicating that their relative rankings should be interpreted as probabilistic patterns rather than clear differences in efficacy.

Moreover, when interpreting these outcome-specific patterns, the potential influence of sensory cues and matched blinding should also be considered. Menthol can induce a cooling sensation, whereas caffeine may produce a distinct bitter taste. These sensory cues may allow participants to infer the assigned condition and weaken blinding. This is particularly relevant for RPE, which is self-reported and may be influenced by expectation or awareness of the intervention. It may also affect effort-dependent performance outcomes, such as self-paced output or time-to-exhaustion performance, because participants’ perceived ergogenic effects could influence pacing strategy, willingness to maintain effort, or power-output regulation. In practice, these sensory cues are difficult to fully eliminate in mouth-rinse trials, even when investigators attempt to use matched placebo solutions. Therefore, even objectively recorded performance outcomes may be indirectly affected when blinding is incomplete.

Overall, mouth rinses containing different ingredients showed certain relative advantages across diverse exercise outcomes. However, given the generally low certainty of evidence for the majority of network comparisons and the absence of stable statistically significant differences among most interventions, these findings should be interpreted as probabilistic and trend-based indications rather than definitive conclusions of superiority.

### 4.2 Practical applications

Because the available evidence primarily involved trained or physically active individuals and cycling- or running-based endurance tasks, the findings are most applicable to these populations and exercise contexts. However, the current evidence does not establish whether elite athletes respond differently from recreationally trained individuals. In the NMA, CAF ranked highest for time to exhaustion, work done during a fixed-time test, and RPE, whereas MEN ranked highest for time to complete a fixed amount of work and mean power; CHO ranked second across all five outcomes. Because no significant differences were observed among the active ingredients, these rankings should be interpreted as probabilistic rather than as evidence of clear superiority. Nevertheless, compared with PLA, CAF and CHO significantly improved time to exhaustion and work done during a fixed-time test, whereas CHO significantly reduced RPE. These findings support considering CAF or CHO for fatigue tolerance and sustained output, and CHO when reducing perceived exertion is relevant.

Although the intervention protocols varied across studies, most used a rinse duration of 5–10 s, and the most frequently investigated concentrations were 6.4% for CHO, 1.2% for CAF, and 0.01% for MEN. Repeated rinsing was also more common than a single rinse. These commonly used protocols may provide practical starting points but should not be regarded as established optimal prescriptions. The available evidence remains insufficient to determine the optimal concentration, number or timing of rinses, event-specific rinsing schedule, or separate strategy for fed and fasted conditions. Therefore, the selected protocol should be tested during training under exercise and feeding conditions similar to those expected in competition.

### 4.3 Strengths and limitations

This study is the first to systematically evaluate the effects of CHO, CAF, and MEN mouth rinses on different exercise performance outcomes and RPE within a cohesive evidence framework, thereby providing cross-ingredient comparative evidence for a literature primarily characterized by comparisons between a single ingredient and PLA. Nevertheless, several limitations should be acknowledged. First, although 57 studies were included, the total sample size was only 609 participants, and many primary studies had small samples and limited statistical power. Second, some direct comparisons were supported by only a few studies, and several network estimates relied mainly on indirect evidence through PLA. Third, variations in participants’ training status, rinse concentration and duration, rinsing timing and frequency, feeding status, task format, and environmental conditions reduced comparability across studies. Because few studies used directly comparable protocols, reliable subgroup analyses could not be conducted. Moreover, different concentrations of the same ingredient were combined within ingredient-level network nodes. Therefore, the present NMA could not determine whether responses differed between elite and recreationally trained individuals, identify the optimal concentration, rinse duration, or rinsing schedule, or establish whether feeding status modified the effects of CHO and CAF or whether event-specific protocols were required. Fourth, the RoB 2 assessment identified concerns related to randomization, period and carryover effects, deviations from intended interventions, and selection of the reported result. The sensory characteristics of CAF and MEN may also have compromised blinding. Fifth, exact paired statistics and within-participant correlations were unavailable for most crossover trials; therefore, *r* = 0.50 was assumed in the primary analysis. Sensitivity analyses using *r* = 0.30 and *r* = 0.70 affected the confidence intervals and the statistical significance of some estimates close to the null, although the effect directions and leading SUCRA rankings remained broadly stable. Finally, CINeMA rated the certainty of most comparisons as low and a few as very low. The findings should therefore be interpreted as trend-based comparative evidence rather than definitive evidence of superiority.

### 4.4 Future directions

Future research should focus on several areas. First, direct comparisons of different mouth-rinse ingredients are needed to reduce the current network’s reliance on indirect evidence. Second, future trials should directly compare different rinse concentrations, rinse durations, rinsing timings, and repetition schedules within the same standardized exercise protocol. Outcomes should be reported in their original units alongside standardized effect estimates to enable ingredient-specific concentration–response analyses and more precise estimation of changes in completion time, work performed, or power output. Training status and feeding status should also be clearly classified and examined as prespecified moderators to determine whether responses differ between elite and recreationally trained individuals or between fed and fasted conditions. Event-specific studies are also needed to determine whether time- or distance-based rinsing schedules should differ across exercise tasks. Third, future trials should report randomization procedures, counterbalancing methods, washout periods, period and carryover effects, blinding checks, and prespecified analysis plans more transparently. Fourth, research populations and exercise contexts should be broadened to include more female participants, high-intensity interval exercise, repeated-sprint exercise, resistance-training tasks, and heat-stress conditions. Neuroimaging, neurophysiological methods, and related physiological indicators should also be integrated to clarify the mechanisms and task specificity of different mouth-rinse ingredients.

## 5 Conclusion

This NMA systematically compared the effects of CHO, CAF, and MEN mouth rinses on exercise performance and RPE. No statistically significant differences were observed among the three ingredients for any of the five outcomes. In comparisons with PLA, CAF and CHO significantly prolonged time to exhaustion and increased work done during a fixed-time test, whereas CHO also significantly reduced RPE. SUCRA rankings placed CAF first for time to exhaustion, work done during a fixed-time test, and RPE, and MEN first for time to complete a fixed amount of work and mean power, with CHO ranking second across all five outcomes. In the absence of significant differences among the three ingredients, these rankings should be interpreted as probabilistic ranking trends rather than evidence of the clear superiority of any single ingredient. Because most included studies involved trained or physically active individuals and predominantly used cycling or running protocols, including time-to-exhaustion and time-trial tasks, the findings may be most applicable to trained or physically active individuals and to exercise settings involving cycling or running.

## Data Availability

The original contributions presented in the study are included in the article/Supplementary material, further inquiries can be directed to the corresponding author.

## Footnotes

1
www.training.cochrane.org/handbook
