# Supplementary material for: Effects of carbohydrate, caffeine and menthol mouth rinses on exercise performance and ratings of perceived exertion: a systematic review and network meta-analysis
Source: Front Nutr. 2026 Aug 19;13:1877453. doi: 10.3389/fnut.2026.1877453 (PMC13534142; doi:10.3389/fnut.2026.1877453)
Supplement: Supplementary file 1 [file Supplementary_file_1.docx]

**Supplementary File S1**

**Table of Contents**

[S1. PRISMA NMA checklist	2](#_Toc7180)

[S2. Search strategy	4](#_Toc2782)

[S3. Detailed Characteristics of Included Studies	6](#_Toc20883)

[S4. Risk-of-Bias Assessment	11](#_Toc11618)

[S5. Evidence Confidence Rating: A CINeMA-Based Evaluation	13](#_Toc3551)

S1. PRISMA NMA checklist

| **Section and Topic** | **Item #** | **Checklist item** | **Location where item is reported** |
| --- | --- | --- | --- |
| **TITLE** | | |  |
| Title | 1 | Identify the report as a systematic review. | 1 |
| **ABSTRACT** | | |  |
| Abstract | 2 | See the PRISMA 2020 for Abstracts checklist. | 1-2 |
| **INTRODUCTION** | | |  |
| Rationale | 3 | Describe the rationale for the review in the context of existing knowledge. | 2-5 |
| Objectives | 4 | Provide an explicit statement of the objective(s) or question(s) the review addresses. | 5-6 |
| **METHODS** | | |  |
| Eligibility criteria | 5 | Specify the inclusion and exclusion criteria for the review and how studies were grouped for the syntheses. | 6-7 |
| Information sources | 6 | Specify all databases, registers, websites, organisations, reference lists and other sources searched or consulted to identify studies. Specify the date when each source was last searched or consulted. | 7 |
| Search strategy | 7 | Present the full search strategies for all databases, registers and websites, including any filters and limits used. | 7 |
| Selection process | 8 | Specify the methods used to decide whether a study met the inclusion criteria of the review, including how many reviewers screened each record and each report retrieved, whether they worked independently, and if applicable, details of automation tools used in the process. | 7 |
| Data collection process | 9 | Specify the methods used to collect data from reports, including how many reviewers collected data from each report, whether they worked independently, any processes for obtaining or confirming data from study investigators, and if applicable, details of automation tools used in the process. | 7 |
| Data items | 10a | List and define all outcomes for which data were sought. Specify whether all results that were compatible with each outcome domain in each study were sought (e.g. for all measures, time points, analyses), and if not, the methods used to decide which results to collect. | 7 |
|  | 10b | List and define all other variables for which data were sought (e.g. participant and intervention characteristics, funding sources). Describe any assumptions made about any missing or unclear information. | 7 |
| Study risk of bias assessment | 11 | Specify the methods used to assess risk of bias in the included studies, including details of the tool(s) used, how many reviewers assessed each study and whether they worked independently, and if applicable, details of automation tools used in the process. | 8 |
| Effect measures | 12 | Specify for each outcome the effect measure(s) (e.g. risk ratio, mean difference) used in the synthesis or presentation of results. | 8 |
| Synthesis methods | 13a | Describe the processes used to decide which studies were eligible for each synthesis (e.g. tabulating the study intervention characteristics and comparing against the planned groups for each synthesis (item #5)). | 8 |
|  | 13b | Describe any methods required to prepare the data for presentation or synthesis, such as handling of missing summary statistics, or data conversions. | 8-9 |
|  | 13c | Describe any methods used to tabulate or visually display results of individual studies and syntheses. | 8-9 |
|  | 13d | Describe any methods used to synthesize results and provide a rationale for the choice(s). If meta-analysis was performed, describe the model(s), method(s) to identify the presence and extent of statistical heterogeneity, and software package(s) used. | 8-9 |
|  | 13e | Describe any methods used to explore possible causes of heterogeneity among study results (e.g. subgroup analysis, meta-regression). | 9 |
|  | 13f | Describe any sensitivity analyses conducted to assess robustness of the synthesized results. | 9-10 |
| Reporting bias assessment | 14 | Describe any methods used to assess risk of bias due to missing results in a synthesis (arising from reporting biases). | 10 |
| Certainty assessment | 15 | Describe any methods used to assess certainty (or confidence) in the body of evidence for an outcome. | 8 |
| **RESULTS** | | |  |
| Study selection | 16a | Describe the results of the search and selection process, from the number of records identified in the search to the number of studies included in the review, ideally using a flow diagram. | 10-11 |
|  | 16b | Cite studies that might appear to meet the inclusion criteria, but which were excluded, and explain why they were excluded. | 10-11 |
| Study characteristics | 17 | Cite each included study and present its characteristics. | 10 |
| Risk of bias in studies | 18 | Present assessments of risk of bias for each included study. | 11-12 |
| Results of individual studies | 19 | For all outcomes, present, for each study: (a) summary statistics for each group (where appropriate) and (b) an effect estimate and its precision (e.g. confidence/credible interval), ideally using structured tables or plots. | 12-19 |
| Results of syntheses | 20a | For each synthesis, briefly summarise the characteristics and risk of bias among contributing studies. | 11-12 |
|  | 20b | Present results of all statistical syntheses conducted. If meta-analysis was done, present for each the summary estimate and its precision (e.g. confidence/credible interval) and measures of statistical heterogeneity. If comparing groups, describe the direction of the effect. | 12-19 |
|  | 20c | Present results of all investigations of possible causes of heterogeneity among study results. | 12-19 |
|  | 20d | Present results of all sensitivity analyses conducted to assess the robustness of the synthesized results. | 20 |
| Reporting biases | 21 | Present assessments of risk of bias due to missing results (arising from reporting biases) for each synthesis assessed. | 20 |
| Certainty of evidence | 22 | Present assessments of certainty (or confidence) in the body of evidence for each outcome assessed. | 8 |
| **DISCUSSION** | | |  |
| Discussion | 23a | Provide a general interpretation of the results in the context of other evidence. | 20-26 |
|  | 23b | Discuss any limitations of the evidence included in the review. | 27 |
|  | 23c | Discuss any limitations of the review processes used. | 27 |
|  | 23d | Discuss implications of the results for practice, policy, and future research. | 27-28 |
| **OTHER INFORMATION** | | |  |
| Registration and protocol | 24a | Provide registration information for the review, including register name and registration number, or state that the review was not registered. | 6 |
|  | 24b | Indicate where the review protocol can be accessed, or state that a protocol was not prepared. | 6 |
|  | 24c | Describe and explain any amendments to information provided at registration or in the protocol. | 6 |
| Support | 25 | Describe sources of financial or non-financial support for the review, and the role of the funders or sponsors in the review. | 28 |
| Competing interests | 26 | Declare any competing interests of review authors. | 28 |
| Availability of data, code and other materials | 27 | Report which of the following are publicly available and where they can be found: template data collection forms; data extracted from included studies; data used for all analyses; analytic code; any other materials used in the review. | 28 |

S2. Search strategy

| Categories | Keywords | Search Results |
| --- | --- | --- |
| PubMed | | |
| *#1* Mouth rinse terms | ("Mouthwashes"[Mesh] OR "mouth rinse"[Title/Abstract] OR "mouth rinsing"[Title/Abstract] OR "mouth-rinse"[Title/Abstract] OR "oral rinse"[Title/Abstract] OR mouthwash[Title/Abstract]) | 11276 |
| *#2* Intervention terms | ("Carbohydrates"[Mesh] OR "Caffeine"[Mesh] OR "Menthol"[Mesh] OR carbohydrate*[Title/Abstract] OR CHO[Title/Abstract] OR maltodextrin[Title/Abstract] OR glucose[Title/Abstract] OR caffeine[Title/Abstract] OR menthol[Title/Abstract]) | 3058876 |
| *#3* Exercise/performance terms | ("Exercise"[Mesh] OR "Sports"[Mesh] OR "Athletes"[Mesh] OR cycling[Title/Abstract] OR running[Title/Abstract] OR exercise[Title/Abstract] OR sport*[Title/Abstract] OR athlete*[Title/Abstract] OR performance[Title/Abstract] OR endurance[Title/Abstract] OR "time trial"[Title/Abstract] OR "time to exhaustion"[Title/Abstract] OR power[Title/Abstract] OR RPE[Title/Abstract] OR "rating of perceived exertion"[Title/Abstract]) | 2864494 |
| *#4* Study design terms | (randomized[Title/Abstract] OR randomised[Title/Abstract] OR crossover[Title/Abstract] OR cross-over[Title/Abstract]  OR placebo[Title/Abstract]) | 1122896 |
|  | *#1* AND *#2* | 1206 |
|  | *#1* AND *#2* AND *#3* | 244 |
|  | *#1* AND *#2* AND *#3* AND *#4* | 161 |
| EMBASE | | |
| *#1* Mouth rinse terms | ("mouth rinse" or "mouth rinsing" or "mouth-rinse" or "oral rinse" or mouthwash).ti,ab. | 49064 |
| *#2* Intervention terms | (carbohydrate* or CHO or maltodextrin or glucose or caffeine or menthol).ti,ab. | 2054769 |
| *#3* Exercise/performance terms | (cycling or running or exercise or sport* or athlete* or performance or endurance or "time trial" or "time to exhaustion" or power or RPE or "rating of perceived exertion").ti,ab. | 4423974 |
| *#4* Study design terms | (randomized or randomised or crossover or cross-over or placebo).ti,ab. | 2345386 |
|  | *#1* AND *#2* | 1989 |
|  | #1 AND #2 AND #3 | 419 |
|  | #1 AND #2 AND #3 AND #4 | 241 |
| Web of Science | | |
| *#1* Mouth rinse terms | TS=("mouth rinse" OR "mouth rinsing" OR "mouth-rinse" OR "oral rinse" OR mouthwash) | 5622 |
| *#2* Intervention terms | TS=(carbohydrate* OR CHO OR maltodextrin OR glucose OR caffeine OR menthol) | 1705038 |
| *#3* Exercise/performance terms | TS=(cycling OR running OR exercise OR sport* OR athlete* OR performance OR endurance OR "time trial" OR "time to exhaustion" OR power OR RPE OR "rating of perceived exertion") | 10834241 |
| *#4* Study design terms | TS=(randomized OR randomised OR crossover OR cross-over OR placebo) | 1414166 |
|  | *#1* AND *#2* | 456 |
|  | #1 AND #2 AND #3 | 331 |
|  | #1 AND #2 AND #3 AND #4 | 194 |
| Cochrane Library | | |
| *#1* Mouth rinse terms | ("mouth rinse" OR "mouth rinsing" OR "mouth-rinse" OR "oral rinse" OR mouthwash):ti,ab,kw | 4402 |
| *#2* Intervention terms | (carbohydrate* OR CHO OR maltodextrin OR glucose OR caffeine OR menthol):ti,ab,kw | 110054 |

| *#3* Exercise/performance terms | (cycling OR running OR exercise OR sport* OR athlete* OR performance OR endurance OR "time trial" OR "time to exhaustion" OR power OR RPE OR "rating of perceived exertion"):ti,ab,kw | 327684 |
| --- | --- | --- |
| *#4* Study design terms | (randomized OR randomised OR crossover OR "cross-over" OR placebo):ti,ab,kw | 1450546 |
|  | *#1* AND *#2* | 275 |
|  | *#1* AND *#2* AND *#3* | 195 |
|  | *#1* AND *#2* AND *#3* AND *#4* | 165 |
| SPORTDiscus | | |
| *#1* Mouth rinse terms | SU("mouth rinse" OR "mouth rinsing" OR "mouth-rinse" OR "oral rinse" OR mouthwash) | 4286 |
| *#2* Intervention terms | SU (carbohydrate* OR CHO OR maltodextrin OR glucose OR caffeine OR menthol) | 885113 |
| *#3* Exercise/performance terms | SU (cycling OR running OR exercise OR sport* OR athlete* OR performance OR endurance OR "time trial" OR "time to exhaustion" OR power OR RPE OR "rating of perceived exertion") | 3955922 |
| *#4* Study design terms | SU (randomized OR randomised OR crossover OR "cross-over" OR placebo) | 1513139 |
|  | *#1* AND *#2* | 1428 |
|  | *#1* AND *#2* AND *#3* | 878 |
|  | *#1* AND *#2* AND *#3* AND *#4* | 567 |

S3. Detailed Characteristics of Included Studies

| Study characteristics | | | Study  design | Population characteristics | | | Intervention characteristics | | | | Outcomes |
| --- | --- | --- | --- | --- | --- | --- | --- | --- | --- | --- | --- |
| Author | Year | Country |  | Status | Sample  size | Age (years) | Rinse composition/concentration (%) | Duration of mouth rinse(s) | Rinsing frequency | Exercise protocol |  |
| Flood et al. | 2017 | UK | Single-blind randomized crossover | Physically active, non-acclimated males | 8 | 26 ± 5 | I:L-menthol,0.01% C:Placebo, apple-flavoured non-calorific sucralose solution | 5 s | 25 mL, pre-exercise and every 10 min | Fixed-RPE cycling to exhaustion in the heat | ①④ |
| Parton et al. | 2021 | UK | Randomized double-blind crossover | Non-acclimated, physically active females and males | 22 (F:11 M:11) | F: 22 ± 2 M: 20 ± 1 | I: L-menthol, 0.01% C: placebo, apple-flavoured sucralose solution. | 10 s | 25 mL, 30 s pre-exercise and every 10 min thereafter | Fixed-RPE cycling to exhaustion in the heat | ①④ |
| Fares & Kayser. | 2011 | Lebanon | Single-blind randomized crossover | Healthy non-athletic males (fed and fasted conditions) | 13 | 21 ± 3 | I: Maltodextrin, 6.4% C: Water. | 10 s | 25 mL every 5 min | Cycling to exhaustion at 60% Wmax | ①⑤ |
| Melo et al. | 2021 | Brazil | Single-blind randomized crossover | Physically active healthy men, recreationally accustomed to cycling | 12 | 22.0 ± 2.8 | I: Caffeine, 1.2% C: Placebo, non-caloric mint essence. | 10 s | Immediately pre-exercise and every 15 min during exercise. | Moderate-intensity cycling to exhaustion at 80% RCP | ①⑤ |
| Fraga et al. | 2017 | Brazil | Randomized double-blind crossover | Endurance-trained males; overnight fasted | 6 | 26 ± 6 | I: Dextrose, 8% C: placebo, non-caloric sweetener. | 10 s | 25 mL, 15 min pre-exercise and every 15 min during exercise | Treadmill running to exhaustion at 85% VO2max | ① |
| Gambassi et al. | 2019 | Brazil | Randomized double-blind crossover | Physically active healthy non-cyclist men | 21 | 22.4 ± 2.7 | I: Maltodextrin, 6.4% C: Placebo, CHO-free lemon-flavoured solution | 10 s | 25 mL at baseline and every 3 min during exercise | Cycling to volitional exhaustion at individual 4 mmol/L lactate threshold | ① |
| Nabuco et al. | 2021 | Brazil | Randomized double-blind crossover | Recreationally trained male cyclists | 10 | 32 ± 3 | I: caffeine, 85 mg/25 mL C: water. | 5 s | 25 mL every 5 min during exercise | Cycling to exhaustion at 75% peak aerobic power | ① |
| Jeffries et al. | 2018 | UK | Randomized crossover | Non-heat-acclimated males; ≥1 year endurance training | 10 | 33 ± 9 | I: L-menthol, 0.01% (0.64 mM) C: placebo, neutral raspberry-flavoured non-calorific mouth rinse | 5 s | Single administration at ~85% of baseline TTE | Cycling to exhaustion at 70% Wmax in the heat (35 °C) | ① |
| Mündel & Jones. | 2010 | UK | Randomized crossover | Healthy, non-heat-acclimated males; cycle-ergometer familiar; overnight fasted | 9 | 25 ± 7 | I: L-menthol, 0.01% (0.64 mM) C: orange-flavoured placebo | 10 s | 25 mL every 10 min during exercise | Cycling to volitional exhaustion at 65% Wmax in the heat (34 °C) | ① |
| Sinclair et al. | 2014 | UK | Randomized crossover | Healthy active male recreational cyclists; 4 h postprandial | 11 | 24.1 ± 3.9 | I: maltodextrin, 6.4% C: water | 5 s/10s | 25 mL every 6 min during exercise | 30-min self-paced cycling time trial | ②④⑤ |
| Bottoms et al. | 2014 | UK | Randomized crossover | Healthy active males; recreationally trained cyclists; 4-h postprandial | 12 | 20.5 ± 0.7 | I: caffeine, 0.032% maltodextrin, 6.4% C: water | 5 s | 25 mL every 6 min during the 30-min trial | 30-min self-paced cycling time trial | ②④⑤ |
| Sinclair & Bottoms. | 2015 | UK | Single-blind randomized crossover | Healthy males; 4-h fasted before trials | 12 | 21.54 ± 1.28 | I: maltodextrin, 6.4% caffeine, 0.032% C: water. | 5 s | 4 × 25 mL, every 6 min | 30-min self-paced arm-crank time trial | ②④⑤ |
| Hawke et al. | 2024 | Canada | Randomized crossover | Trained adolescent male cyclists | 11 | 16.7 ± 1.3 | I: menthol, 0.01% C: placebo，Crystal Light, non-menthol | 5 s | Before each lap; total 6 rinses | 30-min modified variable cycling test in the heat | ②④⑤ |
| Wright & Davison. | 2013 | UK | Randomized double-blind crossover | hysically active male university students; not endurance-trained; 6-h fasted | 7 | 21 ± 1 | I: CHO-electrolyte, 6% and 12% C: placebo, 0% CHO-electrolyte | 5 s | 25 mL at start, 15, 30, and 45 min | 90-min self-paced treadmill run | ②⑤ |
| Rollo et al. | 2010 | UK | Randomized double-blind crossover | Endurance-trained male runners; overnight fasted | 10 | 25 ± 4 | I: CHO-electrolyte, 6.4% C: placebo, color- and taste-matched CHO-free solution | 5 s | 25 mL pre-exercise and at 15, 30, and 45 min | 1-h self-paced treadmill run | ②⑤ |
| Whitham & McKinney. | 2007 | UK | Randomized double-blind crossover | Recreationally active males; 4-h fasted | 7 | 28.5 ± 5.5 | I: maltodextrin, 6% + 3% lemon juice C: placebo, 3% lemon juice | 5 s | Every 6 min during the preload and time trial | 15-min treadmill preload at 65% VO2max + 45-min running time trial | ② |
| Rollo et al. | 2008 | UK | Randomized double-blind crossover | Endurance-trained recreational male runners; overnight fasted | 10 | 23 ± 4 | I: glucose, 6% C: color-, viscosity-, and taste-matched placebo | 5 s | every 5 min during the 30-min run | 30-min self-paced treadmill run at RPE 15 | ② |
| Roriz et al. | 2024 | Portugal | Randomized crossover | Highly trained, non-heat-acclimated male football referees | 9 | 33.4 ± 5.2 | I: menthol, 0.01%; C: placebo, non-caloric berry-flavoured sucralose solution | 10 s | 3 × 25 mL pre-exercise, every 1 min for 3 min before warm-up | SAFT-45 football-specific shuttle running in the heat | ② |
| Carter et al. | 2004 | UK | Randomized crossover | Endurance-trained cyclists | 9 | 24.0 ± 3.8 | I: maltodextrin, 6.4%  C: water. | 5 s | Every 12.5% of trial completed | Fixed-work cycling time trial | ③④⑤ |
| Devenney et al | 2016 | Ireland | Randomized double-blind crossover | Recreationally active males; fed state; cycling-active 3–5 d/week | 12 | 22 ± 7 | I: maltodextrin, 6% and 16% C: placebo, 0% CHO | 5 s | 25 mL after warm-up and every 12.5% of trial completion | Fixed-work cycling time trial in the fed state | ③④⑤ |
| Ispoglou et al. | 2015 | UK | Randomized crossover | Trained male cyclists; postprandial state | 7 | 30 ± 6.7 | I: CHO, 4%, 6%, and 8%  C: non-CHO placebo | 5 s | 25 mL every 12.5% of trial completion | Fixed-work cycling time trial in the postprandial state | ③④⑤ |
| Pottier et al. | 2010 | Belgium | Randomized crossover | Trained subjects; 3-h fasted | 12 | 30.2 ± 5.3 | I:carbohydrate-electrolyte solution, 6%  C: placebo | 5 s | Before exercise and every 12.5% of trial completion | Fixed-work cycling time trial | ③④⑤ |
| Che Muhamed et al | 2014 | Malaysia | Randomized crossover | Ramadan-fasted, trained adolescent male cyclists | 9 | 16 ± 1 | I: carbohydrate solution C: placebo; additional arm: no rinse. | 5 s | 25 mL pre-exercise, at 5, 15, and 25 min of preload, and 15 s before the TT | 30-min cycling preload in hot-humid conditions (32 °C, 75% RH) and 10-km cycling time trial | ③④⑤ |
| Baltazar-Martins & Del Coso. | 2019 | Spain | Randomized double-blind crossover | Well-trained male cyclists; fed state | 16 | 37.6 ± 3.5 | I: mixed carbohydrate drink, 6.4% C: taste-matched placebo, 0.0% carbohydrate | 5 s | 25 mL every 12.5% of trial distance, 7 rinses total | 25.3-km simulated cycling time trial with varying slopes | ③④⑤ |
| Beelen et al. | 2009 | Netherlands | Randomized double-blind crossover | Competitive endurance-trained male cyclists; fed state | 14 | 24 ± 1 | I: maltodextrin, 6.4%  C: water | 10 s | 25 mL at the start and every 12.5% of trial completion | Fixed-work cycling time trial , 2 h after a standardized breakfast | ③④⑤ |
| Trommelen et al | 2015 | Netherlands | Randomized double-blind crossover | Trained male cyclists; fasted and fed state | 14 | 27 ± 6 | I: sucrose, 6.4% C: placebo, 0.6% aspartame | 5 s | 25 mL at the start and every 12.5% of trial completion | Fixed-work cycling time trial in fasted and fed state | ③④ |
| Pires et al | 2018 | Brazil | Randomized double-blind crossover | Recreational male cyclists; fed state | 9 | 36.9 ± 6.4 | I: glucose, 6.4% C: placebo, non-caloric sweetener solution | 10 s | Single 25 mL pre-trial rinse during warm-up | 4-km cycling time trial | ③④ |
| Kulaksız et al | 2016 | Turkey | Randomized double-blind crossover | Recreationally active healthy males; overnight fasted | 9 | 24 ± 2 | I: maltodextrin, 3%, 6%, and 12% C: placebo, 0% maltodextrin | 5 s | 50 mL every 2.5 km | 20-km cycling time trial | ③④ |
| Marinho et al. | 2024 | Brazil | Randomized double-blind crossover | Physically active healthy men | 10 | 24.8 ± 3.7 | I: caffeine mouth rinse, 1.2% C: placebo mouth rinse | 10 s | Single 25 mL pre-TT rinse | 30-min constant-load cycling at 50% Wmax and 10-km cycling time trial | ③④ |
| Clarke et al. | 2017 | UK | Single-blind randomized crossover | Recreational male and pre-menopausal female runners | 15 | F: 43 ± 9 M: 42 ± 10 | I: maltodextrin, 3%, 6%, and 12% C: placebo, 0% maltodextrin | 10 s | 25 mL pre-trial and after each 1 km | 5-km running time trial | ③⑤ |
| Chambers et al. | 2009 | UK | Randomized double-blind crossover | Endurance-trained cyclists; overnight fasted | Study A: 8 Study B: 8 | Study A:29 ± 9 Study B:22 ± 3 | I:Study A,glucose6.4% Study B,maltodextrin6.4% C:artificially sweetened placebo | 10 s | 25 mL at the start and every 12.5% of work completed | Fixed-work cycling time trial | ③⑤ |
| James et al. | 2017 | UK | Randomized double-blind crossover | Male cyclists; overnight fasted | 11 | 40 ± 8 | I: maltodextrin, 7% and 14% C: taste-matched placebo | 5 s | Every 12.5% of trial completion | Fixed-work cycling time trial | ③⑤ |
| Ali et al. | 2017 | New Zealand | Randomized crossover | Moderately trained male cyclists and triathletes; glycogen-reduced/overnight fasted | 9 | 32.7 ± 13.0 | I: CHO mouth rinse, 15% C: placebo mouth rinse, 0% CHO (aspartame) | 8 s | 0.33 mL·kg⁻¹ every 12.5% of exercise completed | Fixed-work cycling time trial | ③⑤ |
| Figueiredo et al. | 2021 | Brazil | Randomized double-blind crossover | Recreational runners, CYP1A2 C-allele carriers | 10 | 30.1 ± 6.4 | I: caffeine, 1.2% (300 mg/25 mL) C: microcrystalline cellulose in water. | 10 s | Single 25 mL pre-run rinse | 10-km running time trial | ③⑤ |
| Stevens et al. | 2017 | Australia | Randomized crossover | Trained male runners | 11 | 30 ± 9 | I: facial water spray + menthol mouth rinse C: No rinsing | 5 s | pre-exercise | 3-km running time trial | ③⑤ |
| Silva et al. | 2016 | Brazil | Randomized double-blind crossover | Healthy males; FED, 12-h FAST, and DEP + 12-h FAST conditions | 8 | 31.5 ± 7.3 | I: maltodextrin, 6.4% C: placebo. | 10 s | Every 12.5% of trial completion | 20-km cycling time trial. | ③ |
| Gam et al. | 2013 | Australia | Randomized crossover | Male cyclists | 10 | 29.0 ± 9.1 | I: maltodextrin, 6.4% C: water | 5 s | Every 12.5% of trial completion | 1000-kJ cycling time trial | ③ |
| Barbosa et al. | 2020 | Brazil | Randomized double-blind crossover | Recreationally endurance-trained men | 7 | 24.6 ± 11.5 | I: anhydrous caffeine, 0.6% (300 mg/50 mL) C: placebo, microcrystalline cellulose. | 10 s | Single 50 mL pre-trial rinse | 800-m running time trial | ③ |
| Boat et al. | 2021 | UK | Randomized double-blind crossover | Endurance recreationally trained male cyclists; 4-h postprandial | 15 | 22.4 ± 2.56 | I: caffeine, 40 mg/25 mL C: taste-, texture-, and color-matched placebo. | 10 s | Pre-trial and every 2 km during the trial | 10-km cycling time trial | ③ |
| Doering et al. | 2014 | Australia | Randomized double-blind crossover | Well-trained male cyclists | 10 | 32.9 ± 7.5 | I: anhydrous caffeine, 35 mg/25 mL C: placebo, de-carbonated non-caffeinated diet cola beverage | 10 s | 25 mL, 30 s pre-trial and every 12.5% of trial completion (8 rinses total) | Fixed-work cycling time trial | ③ |
| Stevens et al. | 2016 | Australia | Randomized crossover | Moderately trained, heat-acclimatized male runners | 11 | 29 ± 9 | I: L-menthol, 0.01% C: no intervention; additional arm: ice slurry, 7.5 g/kg BM | 5 s | 25 mL during the run, at 0.2 km of every 1 km | 5-km running time trial in the heat (33 °C) on a non-motorized treadmill | ③ |
| Gavel et al. | 2024 | Canada | Randomized crossover | Trained male cyclists | 12 | 24.2 ± 3.2 | I: L-menthol, 0.1% + cold water; L-menthol, 0.1% + thermoneutral water.  C: cold water; thermoneutral water. | 5 s | 25 mL pre-trial and every 5 km | 20-km cycling time trial in thermoneutral conditions | ③ |
| Jeffers et al. | 2015 | UK | Randomized double-blind crossover | Moderate- to well-trained male cyclists/triathletes; regional/national competitors; TT-experienced; 4-h fasted | 9 | 33.2 ± 7.4 | I: maltodextrin, 6.4% C: water | 5 s | 25 mL immediately before and every 7.5 min during the 45-min preloadthen immediately before and halfway through the 15-min TT | 45-min cycling preload at 70% Wmax + 15-min cycling time trial | ④ |
| Lane et al. | 2013 | Australia | Randomized crossover | Competitive endurance-trained male triathletes; fed and overnight-fasted conditions. | 12 | 28 ± 5 | I: maltodextrin, 10% C: taste-matched placebo. | 10 s | Pre-trial and every 12.5% of total time during the ride | 60-min simulated cycling time trial | ④ |
| Kizzi et al. | 2016 | UK | Single-blind randomized crossover | Recreationally active males | 8 | 23 ± 2 | I: caffeine, 2% C: taste-matched non-caloric placebo | 10 s | 25 mL before the sprint test and between sprints; 6 rinses total | Five 6-s sprint cycling bouts with 24-s active recovery, following glycogen depletion. | ④ |
| Gough et al. | 2022 | UK | Randomized double-blind crossover | Male recreational soccer players; high-CHO meal 2 h pre-trial | 9 | 21 ± 3 | I: caffeine, 6 mg·kg⁻¹ + 10%; caffeine C: water | 10 s | Single 25 mL rinse immediately before the second RSAT (75 min of SAFT90) | SAFT90 + RSAT (6 × 6-s sprints, 24-s recovery) | ④ |
| Karayiğit et al. | 2017 | Turkey | Randomized double-blind crossover | Physically active healthy males; fasted state | 10 | 20.50 ± 1.58 | I: caffeine, 2% w/v C: water | 10 s | 25 mL immediately before and every 60 s during 5-min warm-up; 6 rinses total. | 30-s Wingate anaerobic test | ④ |
| Marinho et al. | 2020 | Brazil | Randomized double-blind crossover | Healthy men | 10 | 24.8 ± 3.7 | I: caffeine, 1.2% (25 mL; 300 mg) C: mint syrup without caffeine | 10 s | Single pre-test rinse | 30-s Wingate anaerobic test | ④ |
| Watson et al. | 2014 | UK | Randomized double-blind crossover | Endurance-trained males | 10 | 21 ± 2 | T: glucose, 6.4%  C: placebo. | 10 s | Immediately pre-exercise and at regular intervals during exercise | 1-h cycling time trial in the heat (30 °C, 60% RH) | ④⑤ |
| Clarke et al. | 2017 | UK | Randomized crossover | Healthy males; intermediate resistance-trained; 11-h overnight fast; tested at 07:30 | 12 | 23 ± 3 | I: maltodextrin, 6% C: water, orange-flavoured placebo | 10 s | 25 mL before each exercise | CMJ, isometric mid-thigh pull, 10-m sprint, bench press and back squat repetitions to failure at 60% 1-RM | ⑤ |
| Simpson et al. | 2018 | USA | Randomized double-blind crossover | Trained men; overnight fasted | 7 | 28.2 ± 6.8 | I: maltodextrin, 6.4% C: flavor-matched non-caloric sport beverage placebo | 10 s | 25 mL before each sprint set (at 4:30 of each 5-min steady-state segment); 6 rinses total | Intermittent cycling | ⑤ |
| Přibyslavská et al. | 2015 | USA | Randomized double-blind crossover | Collegiate female soccer players; overnight fasted | 11 | 20 ± 1 | I: maltodextrin, 6% C: taste- and colour-matched placebo | 15 s | 20 mL, 5 min pre-exercise and during each 1.5-min recovery period | 3 × 5-min 3-v-3 soccer scrimmage bouts with 1VJ, 4VJ, 18-m sprint, and 72-m shuttle run | ⑤ |
| Bailey et al. | 2025 | UK | Randomized double-blind crossover | Highly trained/elite adolescent swimmers | 11 | 17.8 ± 3.1 | I: maltodextrin, 6.4% C: taste- and colour-matched placebo | 10 s | 25 mL before each sprint | 4 × 50-m maximal-effort interval swimming with 30-s rest | ⑤ |
| Phillips et al. | 2014 | UK | Randomized double-blind crossover | Physically active males; postprandial state | 12 | 23.1 ± 3.0 | I: maltodextrin, 6% w/v C: placebo solution | 5 s | 8 × 25 mL during a 7-min warm-up; every 2 min, with final rinse before sprint | 30-s cycle sprint (Wingate-type) | ⑤ |
| Karayigit et al. | 2021 | Turkey | Randomized double-blind crossover | Resistance-trained females | 16 | 20 ± 1 | I: CHO, 6%, 12%, and 18% C: water | 10 s | Single 25 mL pre-exercise rinse | Bench press 1-RM + bench press repetitions to failure at 40% 1-RM | ⑤ |
| Clarke et al. | 2015 | UK | Randomized double-blind crossover | Recreationally resistance-trained males | 15 | 21 ± 2 | I: carbohydrate, 6%; caffeine, 1.2%;  C: water | 10 s | Single 25 mL pre-exercise rinse | Bench press 1-RM + bench press repetitions to failure at 60% 1-RM | ⑤ |
| Dolan et al. | 2017 | USA | Randomized double-blind crossover | Collegiate male lacrosse players (NCAA Division II); in-season; overnight fasted | 10 | 19.9 ± 1.3 | I: CHO, 6%; CAF, 1.2% C: flavored water | 10 s | Single 25 mL pre-exercise rinse | Yo-Yo Intermittent Recovery Test Level 1 | ⑤ |

Note:M：male , F:female , T:intervention , C:control , ①:Time to exhaustion ,②:work done during a fixed-time test ,③:Completion time for a fixed workload ,④:Mean power output ,⑤:Rating of perceived exertion

S4. Risk-of-Bias Assessment

**Figure S4.1.** Detailed risk-of-bias judgments for individual studies on objective exercise performance outcomes.

***Note:*** Green indicates low risk of bias, yellow indicates some concerns, and red indicates high risk of bias. The assessment was conducted using the revised Cochrane risk-of-bias tool for randomized crossover trials. Objective exercise performance outcomes included time to exhaustion, work done during a fixed-time test, time to complete a fixed amount of work, and mean power output. The columns from left to right represent bias arising from the randomization process, bias arising from period and carryover effects, bias due to deviations from intended interventions, bias due to missing outcome data, bias in measurement of the outcome, bias in selection of the reported result, and overall risk of bias.

**Figure S4.2.** Detailed risk-of-bias judgments for individual studies on the subjective outcome of ratings of perceived exertion.

***Note:*** Green indicates low risk of bias, yellow indicates some concerns, and red indicates high risk of bias. The assessment was conducted using the revised Cochrane risk-of-bias tool for randomized crossover trials. Subjective exercise performance outcomes referred to ratings of perceived exertion. The columns from left to right represent bias arising from the randomization process, bias arising from period and carryover effects, bias due to deviations from intended interventions, bias due to missing outcome data, bias in measurement of the outcome, bias in selection of the reported result, and overall risk of bias.

S5. Evidence Confidence Rating: A CINeMA-Based Evaluation

| **Time to exhaustion** | | | | | | | |
| --- | --- | --- | --- | --- | --- | --- | --- |
| Comparison | Within-study bias | Reporting bias | Indirectness | Imprecision | Heterogeneity | Incoherence | Confidence rating |
| CAF vs PLA | Major concerns | Low risk | No concerns | Major concerns | No concerns | No concerns | Very low |
| CHO vs PLA | Some concerns | Low risk | No concerns | Major concerns | No concerns | No concerns | Low |
| MEN vs PLA | Some concerns | Low risk | No concerns | Major concerns | No concerns | No concerns | Low |
| CAF vs CHO | Some concerns | Low risk | No concerns | Major concerns | No concerns | No concerns | Low |
| CAF vs MEN | Some concerns | Low risk | No concerns | Major concerns | No concerns | No concerns | Low |
| CHO vs MEN | Some concerns | Low risk | No concerns | Major concerns | No concerns | No concerns | Low |
| **Work done during a fixed-time test** | | | | | | | |
| CAF vs PLA | No concerns | Low risk | No concerns | No concerns | Major concerns | No concerns | Low |
| CHO vs PLA | No concerns | Low risk | No concerns | Major concerns | No concerns | No concerns | Low |
| MEN vs PLA | Some concerns | Low risk | No concerns | Major concerns | No concerns | No concerns | Low |
| CAF vs CHO | No concerns | Low risk | No concerns | Major concerns | No concerns | No concerns | Low |
| CAF vs MEN | Some concerns | Low risk | No concerns | Major concerns | No concerns | No concerns | Low |
| CHO vs MEN | Some concerns | Low risk | No concerns | Major concerns | No concerns | No concerns | Low |
| **Completion time for a fixed workload** | | | | | | | |
| CAF vs PLA | No concerns | Low risk | No concerns | Major concerns | No concerns | No concerns | Low |
| CHO vs PLA | No concerns | Low risk | No concerns | Major concerns | No concerns | No concerns | Low |
| MEN vs PLA | Some concerns | Low risk | No concerns | Major concerns | No concerns | No concerns | Low |
| CAF vs CHO | No concerns | Low risk | No concerns | Major concerns | No concerns | No concerns | Low |
| CAF vs MEN | No concerns | Low risk | No concerns | Major concerns | No concerns | No concerns | Low |
| CHO vs MEN | Some concerns | Low risk | No concerns | Major concerns | No concerns | No concerns | Low |
| **Mean power output** | | | | | | | |
| CAF vs PLA | No concerns | Low risk | No concerns | Major concerns | No concerns | No concerns | Low |
| CHO vs PLA | No concerns | Low risk | No concerns | Major concerns | No concerns | No concerns | Low |
| MEN vs PLA | No concerns | Low risk | No concerns | Major concerns | No concerns | No concerns | Low |
| CAF vs CHO | No concerns | Low risk | No concerns | Major concerns | No concerns | No concerns | Low |
| CAF vs MEN | No concerns | Low risk | No concerns | Major concerns | No concerns | No concerns | Low |
| CHO vs MEN | No concerns | Low risk | No concerns | Major concerns | No concerns | No concerns | Low |
| **Rating of perceived exertion** | | | | | | | |
| CAF vs PLA | No concerns | Low risk | No concerns | Major concerns | No concerns | No concerns | Low |
| CHO vs PLA | No concerns | Low risk | No concerns | Major concerns | No concerns | No concerns | Low |
| MEN vs PLA | No concerns | Low risk | No concerns | Major concerns | No concerns | No concerns | Low |
| CAF vs CHO | Some concerns | Low risk | No concerns | Major concerns | No concerns | No concerns | Low |
| CAF vs MEN | Some concerns | Low risk | No concerns | Major concerns | No concerns | No concerns | Low |
| CHO vs MEN | Some concerns | Low risk | No concerns | Major concerns | No concerns | No concerns | Low |

*****Note*:**** This table presents the evidence quality assessment of the included studies based on the **CINeMA (Confidence in Network Meta-Analysis)** framework, which evaluates six key domains:

1.**Within-study bias** – Risk of bias arising from the design and conduct of the individual studies;

2.**Reporting bias** – Potential for selective outcome reporting;

3.**Indirectness** – Degree of relevance between the interventions, population, and outcomes versus the research question;

4.**Imprecision** – Uncertainty due to small sample sizes or wide confidence intervals;

5.**Heterogeneity** – Variability in effect estimates across studies;

**6.Incoherence** – Discrepancies between direct and indirect evidence within the network. Each domain is rated as **“No concerns”**, **“Some concerns”**, or **“Major concerns”**, and the overall confidence in the evidence is summarized as **High**, **Moderate**, **Low**, or **Very Low**.
