# Supplementary material for: Effects of carbohydrate, caffeine and menthol mouth rinses on exercise performance and ratings of perceived exertion: a systematic review and network meta-analysis
Source: Front Nutr. 2026 Aug 19;13:1877453. doi: 10.3389/fnut.2026.1877453 (PMC13534142; doi:10.3389/fnut.2026.1877453)
Supplement: Supplementary file 2 [file Supplementary_file_2.docx]

S6. Pairwise Comparisons and Network Meta-Analysis

S6.1 Results of Pairwise and Network Meta-Analyses and Meta-Regression on Time to Exhaustion Outcomes

S6.1.1 Pairwise Meta-analysis Results

[1] CHO vs PLA（n=3）

[2] CAF VS PLA（n=2）

[3] MEN vs PLA（n=5）

S6.1.2 Ranking Probabilities

| **Rank** | **CAF** | **CHO** | **MEN** | **PLA** |
| --- | --- | --- | --- | --- |
| Best | 66.2 | 28.2 | 5.6 | 0.0 |
| 2nd | 23.4 | 51.4 | 25.2 | 0.0 |
| 3rd | 10.0 | 20.0 | 65.9 | 4.1 |
| Worst | 0.4 | 0.4 | 3.3 | 95.8 |

S6.1.3 Cumulative Ranking Probabilities

****Note**:** This figure presents the cumulative ranking curve (SUCRA) from the network meta-analysis, illustrating the cumulative probabilities of each intervention occupying various ranks. The closer the curve is to the top-left corner, the more effective the intervention is considered.

S6.1.4 Rank Probability Plot

**Note:** This figure displays the ranking probability plot from the network meta-analysis, showing the independent probability distribution of each intervention at each possible rank. A higher curve indicates a greater likelihood that the corresponding intervention occupies that specific rank.

S6.1.5 SUCRA

| **Treatment** | **SUCRA** | **PrBest** | **MeanRank** |
| --- | --- | --- | --- |
| CAF | 85.1 | 66.2 | 1.4 |
| CHO | 69.1 | 28.2 | 1.9 |
| MEN | 44.3 | 5.6 | 2.7 |
| PLA | 1.4 | 0.0 | 4.0 |

S6.1.6 Forest Plot

S6.1.7 Publication Bias

| **Test Type** | **Model** | **β₁ / Kendall's Score** | **Standard Error (SE)** | **z-Value** | **p-Value** |
| --- | --- | --- | --- | --- | --- |
| Egger's Regression | Random-effects (REML) | -1.35 | 1.892 | -0.72 | 0.4741 |

S6.1.8 Funnel Plot

S6.2 Results of Pairwise and Network Meta-Analyses and Meta-Regression on Work Done During a Fixed-Time Test Outcomes

S6.2.1 Pairwise Meta-analysis Results

[1]CHO vs PLA（n=7）

[2] CAF vs PLA（n=2）

[3] MEN vs PLA（n=2）

S6.2.2 Ranking Probabilities

| **Rank** | **CAF** | **CHO** | **MEN** | **PLA** |
| --- | --- | --- | --- | --- |
| Best | 62.9 | 17.6 | 19.5 | 0.0 |
| 2nd | 24.9 | 53.6 | 21.4 | 0.1 |
| 3rd | 11.8 | 28.6 | 47.3 | 12.3 |
| Worst | 0.4 | 0.1 | 11.8 | 87.6 |

S6.2.3 Cumulative Ranking Probabilities

****Note**:** This figure presents the cumulative ranking curve (SUCRA) from the network meta-analysis, illustrating the cumulative probabilities of each intervention occupying various ranks. The closer the curve is to the top-left corner, the more effective the intervention is considered.

S6.2.4 Rank Probability Plot

**Note:** This figure displays the ranking probability plot from the network meta-analysis, showing the independent probability distribution of each intervention at each possible rank. A higher curve indicates a greater likelihood that the corresponding intervention occupies that specific rank.

S6.2.5 SUCRA

| **Treatment** | **SUCRA** | **PrBest** | **MeanRank** |
| --- | --- | --- | --- |
| CAF | 83.4 | 62.9 | 1.5 |
| CHO | 62.9 | 17.6 | 2.1 |
| MEN | 49.5 | 19.5 | 2.5 |
| PLA | 4.2 | 0.0 | 3.9 |

S6.2.6 Forest Plot

S6.2.7 Publication Bias

| **Test Type** | **Model** | **β₁ / Kendall's Score** | **Standard Error (SE)** | **z-Value** | **p-Value** |
| --- | --- | --- | --- | --- | --- |
| Egger's Regression | Random-effects (REML) | 0.69 | 3.176 | 0.22 | 0.8281 |

S6.2.8 Funnel Plot

S6.3 Results of Pairwise and Network Meta-Analyses and Meta-Regression on Time to Complete a Fixed Amount of Work Outcomes

S6.3.1 Pairwise Meta-analysis Results

[1] CHO vs PLA（n=17）

| **Type** | **Variable** | **Coefficient** | **Std. err.** | **t** | **P>\|t\|** | **[95% conf. interval]** |
| --- | --- | --- | --- | --- | --- | --- |
| CHO | Age | .0041872 | .0082907 | 0.51 | 0.614 | -.0120623, .0204366 |
|  | _cons | -.2583057 | .2637908 | -0.98 | 0.327 | -.7753261,.2587148 |
|  | Sample Size | .0107555 | .0267051 | 0.40 | 0.687 | -.0415855,.0630964 |
|  | _cons | -.2519451 | .3104974 | -0.81 | 0.417 | -.8605089,.3566186 |
|  | **Time spent rinsing** | .0335692 | .0319211 | 1.05 | 0.293 | -.028995,.0961333 |
|  | _cons | -.3618984 | .232462 | -1.56 | 0.120 | -.8175156,.0937189 |

Meta-regression Analysis

[2] CAF vs PLA（n=5）

[3] MEN vs PLA（n=3）

S6.3.2 Ranking Probabilities

| **Rank** | **CAF** | **CHO** | **MEN** | **PLA** |
| --- | --- | --- | --- | --- |
| Best | 6.4 | 29.5 | 63.8 | 0.3 |
| 2nd | 15.6 | 55.3 | 19.7 | 9.4 |
| 3rd | 23.8 | 13.3 | 8.6 | 54.3 |
| Worst | 54.3 | 1.9 | 7.9 | 36.0 |

S6.3.3 Cumulative Ranking Probabilities

****Note**:** This figure presents the cumulative ranking curve (SUCRA) from the network meta-analysis, illustrating the cumulative probabilities of each intervention occupying various ranks. The closer the curve is to the top-left corner, the more effective the intervention is considered.

S6.3.4 Rank Probability Plot

**Note:** This figure displays the ranking probability plot from the network meta-analysis, showing the independent probability distribution of each intervention at each possible rank. A higher curve indicates a greater likelihood that the corresponding intervention occupies that specific rank.

S6.3.5 SUCRA

| **Treatment** | **SUCRA** | **PrBest** | **MeanRank** |
| --- | --- | --- | --- |
| CAF | 24.7 | 6.4 | 3.3 |
| CHO | 70.8 | 29.5 | 1.9 |
| MEN | 79.8 | 63.8 | 1.6 |
| PLA | 24.7 | 0.3 | 3.3 |

S6.3.6 Forest Plot

S6.3.7 Publication Bias

| **Test Type** | **Model** | **β₁ / Kendall's Score** | **Standard Error (SE)** | **z-Value** | **p-Value** |
| --- | --- | --- | --- | --- | --- |
| Egger's Regression | Random-effects (REML) | -0.44 | 1.699 | -0.26 | 0.7950 |

S6.3.8 Funnel Plot

S6.4 Results of Pairwise and Network Meta-Analyses and Meta-Regression on Mean Power Outcomes

S6.4.1 Pairwise Meta-analysis Results

[1]CHO vs PLA（n=16）

| **Type** | **Variable** | **Coefficient** | **Std. err.** | **t** | **P>\|t\|** | **[95% conf. interval]** |
| --- | --- | --- | --- | --- | --- | --- |
| CHO | Age | -.0030896 | .0128332 | -0.24 | 0.810 | -.0282423,.0220631 |
|  | _cons | .2243986 | .3478548 | 0.65 | 0.519 | -.4573842,.9061815 |
|  | Sample Size | .020798 | .032345 | 0.64 | 0.520 | -.042597,.0841931 |
|  | _cons | -.0972638 | .3807887 | -0.26 | 0.798 | -.843596,.6490684 |
|  | **Time spent rinsing** | -.0197929 | .0347509 | -0.57 | 0.569 | -.0879033,.0483175 |
|  | _cons | .267051 | .2311938 | 1.16 | 0.248 | -.1860805,.7201825 |

Meta-regression Analysis

[2] CAF vs PLA（n=7）

[3] MEN vs PLA（n=4）

S6.4.2 Ranking Probabilities

| **Rank** | **CAF** | **CHO** | **MEN** | **PLA** |
| --- | --- | --- | --- | --- |
| Best | 21.4 | 18.4 | 60.1 | 0.1 |
| 2nd | 33.9 | 45.2 | 18.8 | 2.1 |
| 3rd | 31.0 | 33.3 | 13.9 | 21.8 |
| Worst | 13.7 | 3.1 | 7.2 | 76.1 |

S6.4.3 Cumulative Ranking Probabilities

****Note**:** This figure presents the cumulative ranking curve (SUCRA) from the network meta-analysis, illustrating the cumulative probabilities of each intervention occupying various ranks. The closer the curve is to the top-left corner, the more effective the intervention is considered.

S6.4.4 Rank Probability Plot

**Note:** This figure displays the ranking probability plot from the network meta-analysis, showing the independent probability distribution of each intervention at each possible rank. A higher curve indicates a greater likelihood that the corresponding intervention occupies that specific rank.

S6.4.5 SUCRA

| **Treatment** | **SUCRA** | **PrBest** | **MeanRank** |
| --- | --- | --- | --- |
| CAF | 54.4 | 21.4 | 2.4 |
| CHO | 59.7 | 18.4 | 2.2 |
| MEN | 77.3 | 60.1 | 1.7 |
| PLA | 8.7 | 0.1 | 3.7 |

S6.4.6 Forest Plot

S6.4.7 Publication Bias

| **Test Type** | **Model** | **β₁ / Kendall's Score** | **Standard Error (SE)** | **z-Value** | **p-Value** |
| --- | --- | --- | --- | --- | --- |
| Egger's Regression | Random-effects (REML) | -1.41 | 1.867 | -0.76 | 0.4488 |

S6.4.8 Funnel Plot

S6.5 Results of Pairwise and Network Meta-Analyses and Meta-Regression on Ratings of Perceived Exertion Outcomes

S6.5.1 Pairwise Meta-analysis Results

[1] CHO vs PLA（n=27）

| **Type** | **Variable** | **Coefficient** | **Std. err.** | **t** | **P>\|t\|** | **[95% conf. interval]** |
| --- | --- | --- | --- | --- | --- | --- |
| CHO | Age | .0006644 | .0073219 | 0.09 | 0.928 | -.0136862,.015015 |
|  | _cons | -.1521963 | .1972392 | -0.77 | 0.440 | -.538778,.2343854 |
|  | Sample Size | .0059292 | .0231516 | 0.26 | 0.798 | -.0394471,.0513054 |
|  | _cons | -.2038535 | .2751722 | -0.74 | 0.459 | -.743181,.3354741 |
|  | **Time spent rinsing** | -.0352823 | .0208053 | -1.70 | 0.090 | -.0760599,.0054953 |
|  | _cons | .1405181 | .1724173 | 0.81 | 0.415 | -.1974136,.4784498 |

Meta-regression Analysis

[2] CAF vs PLA（n=6）

[3] MEN vs PLA（n=2）

S6.5.2 Ranking Probabilities

| **Rank** | **CAF** | **CHO** | **MEN** | **PLA** |
| --- | --- | --- | --- | --- |
| Best | 47.4 | 31.5 | 20.9 | 0.2 |
| 2nd | 32.2 | 50.7 | 12.2 | 4.9 |
| 3rd | 15.5 | 17.2 | 17.6 | 49.7 |
| Worst | 4.9 | 0.6 | 49.3 | 45.2 |

S6.5.3 Cumulative Ranking Probabilities

**Note:** This figure presents the cumulative ranking curve (SUCRA) from the network meta-analysis, illustrating the cumulative probabilities of each intervention occupying various ranks. The closer the curve is to the top-left corner, the more effective the intervention is considered.

S6.5.4 Rank Probability Plot

**Note:** This figure displays the ranking probability plot from the network meta-analysis, showing the independent probability distribution of each intervention at each possible rank. A higher curve indicates a greater likelihood that the corresponding intervention occupies that specific rank.

| **Treatment** | **SUCRA** | **PrBest** | **MeanRank** |
| --- | --- | --- | --- |
| CAF | 74.0 | 47.4 | 1.8 |
| CHO | 71.1 | 31.5 | 1.9 |
| MEN | 34.9 | 20.9 | 3.0 |
| PLA | 20.0 | 0.2 | 3.4 |

S6.5.5 SUCRA

S6.5.6 Forest Plot

S6.5.7 Publication Bias

| **Test Type** | **Model** | **β₁ / Kendall's Score** | **Standard Error (SE)** | **z-Value** | **p-Value** |
| --- | --- | --- | --- | --- | --- |
| Egger's Regression | Random-effects (REML) | -0.16 | 1.496 | -0.11 | 0.9161 |

S6.5.8 Funnel Plot

S7. Sensitivity Analyses Using Alternative Within-Participant Correlation Coefficients

S7.1. Sensitivity Analysis Assuming r=0.30

S7.1.1 Network meta-analysis results and SUCRA rankings assuming a within-participant correlation coefficient of r=0.30

| Outcome | CAF vs PLA, SMD (95% CI) | CHO vs PLA, SMD (95% CI) | MEN vs PLA, SMD (95% CI) | CHO vs CAF SMD (95% CI) | MEN vs CAF SMD (95% CI) | MEN vs CHO SMD (95% CI) | SUCRA ranking |
| --- | --- | --- | --- | --- | --- | --- | --- |
| Time to exhaustion | **0.58 (0.07,1.09)** | **0.43 (0.06, 0.81)** | 0.28 (−0.06, 0.61) | −0.14 (−0.78, 0.49) | −0.30 (−0.91, 0.31) | −0.16 (−0.66, 0.34) | CAF 82.9% > CHO 68.6% > MEN 45.9% > PLA 2.6% |
| Work done during a fixed-time test | **0.49 (0.05, 0.93)** | **0.36 (0.08, 0.65)** | 0.28 (−0.25, 0.80) | −0.13 (−0.57, 0.31) | −0.21 (−0.90, 0.47) | −0.08 (−0.68, 0.51) | CAF 81.1% > CHO 63.2% > MEN 50.0% > PLA 5.7% |
| Time to complete a fixed amount of work | 0.03 (−0.30, 0.35) | −0.13 (−0.31, 0.04) | −0.21 (−0.61, 0.19) | −0.16 (−0.52, 0.21) | −0.24 (−0.75, 0.28) | −0.08 (−0.52, 0.36) | MEN 77.0% > CHO 69.4% > CAF 27.3% > PLA 26.4% |
| Mean power | 0.12 (−0.15, 0.39) | 0.13 (−0.04, 0.31) | 0.23 (−0.13, 0.60) | 0.01 (−0.29, 0.31) | 0.11 (−0.35, 0.56) | 0.10 (−0.31, 0.50) | MEN 75.1% > CHO 59.5% > CAF 53.5% > PLA 11.9% |
| Ratings of perceived exertion | −0.16 (−0.41, 0.08) | **−0.14 (−0.28, -0.01)** | 0.00 (−0.49, 0.49) | 0.02 (−0.23, 0.28) | 0.16 (−0.39, 0.72) | 0.14 (−0.37, 0.65) | CAF 73.0% > CHO 70.8% > MEN 35.8% > PLA 20.3% |

S7.1.2 Pairwise Meta-Analysis Results Assuming a Within-Participant Correlation Coefficient of r=0.30

| Outcome | Comparison | SMD( 95% CI) | I^2^ (%) | P value |
| --- | --- | --- | --- | --- |
| Time to exhaustion | CHO vs PLA | **0.43 (0.06, 0.81)** | 0.00 | **0.02** |
|  | CAF vs PLA | **0.58 (0.07, 1.09)** | 0.00 | **0.03** |
|  | MEN vs PLA | 0.28 (−0.06, 0.61) | 0.00 | 0.11 |
| Work done during a fixed-time test | CHO vs PLA | **0.36 (0.08, 0.64)** | 0.00 | **0.01** |
|  | CAF vs PLA | **0.56 (0.07, 1.05)** | 0.00 | **0.02** |
|  | MEN vs PLA | 0.28 (−0.25, 0.80) | 0.00 | 0.30 |
| Time to complete a fixed amount of work | CHO vs PLA | −0.13 (−0.31, 0.04) | 0.00 | 0.13 |
|  | CAF vs PLA | 0.03 (−0.30, 0.35) | 0.00 | 0.87 |
|  | MEN vs PLA | −0.21 (−0.61, 0.19) | 0.00 | 0.30 |
| Mean power | CHO vs PLA | 0.15 (−0.03, 0.32) | 0.00 | 0.11 |
|  | CAF vs PLA | 0.14 (−0.30, 0.58) | 57.96 | 0.54 |
|  | MEN vs PLA | 0.23 (−0.13, 0.60) | 0.00 | 0.21 |
| Ratings of perceived exertion | CHO vs PLA | −0.13 (−0.27, 0.00) | 0.00 | 0.05 |
|  | CAF vs PLA | −0.15 (−0.51, 0.22) | 40.16 | 0.43 |
|  | MEN vs PLA | 0.00 (−0.49, 0.49) | 0.00 | 1.00 |

S7.2. Sensitivity Analysis Assuming r=0.70

S7.2.1 Network meta-analysis results and SUCRA rankings assuming a within-participant correlation coefficient of r=0.70

| Outcome | CAF vs PLA, SMD (95% CI) | CHO vs PLA, SMD (95% CI) | MEN vs PLA, SMD (95% CI) | CHO vs CAF SMD (95% CI) | MEN vs CAF SMD (95% CI) | MEN vs CHO SMD (95% CI) | SUCRA ranking |
| --- | --- | --- | --- | --- | --- | --- | --- |
| Time to exhaustion | **0.56 (0.20, 0.92)** | **0.42 (0.16, 0.67)** | **0.27 (0.04, 0.49)** | −0.14 (−0.58, 0.29) | −0.29 (−0.71, 0.13) | −0.15 (−0.49, 0.19) | CAF 88.5% > CHO 69.5% > MEN 41.6% > PLA 0.4% |
| Work done during a fixed-time test | **0.47 (0.17, 0.77)** | **0.35 (0.16, 0.54)** | 0.26 (−0.09, 0.61) | −0.12 (−0.41, 0.17) | −0.21 (−0.67, 0.25) | −0.09 (−0.49, 0.31) | CAF 86.7% > CHO 62.7% > MEN 48.1% > PLA 2.5% |
| Time to complete a fixed amount of work | 0.03 (−0.19, 0.24) | **−0.13 (−0.24, −0.01)** | −0.21 (−0.47, 0.06) | −0.15 (−0.39, 0.09) | −0.23 (−0.57, 0.11) | −0.08 (−0.37, 0.21) | MEN 85.0% > CHO 72.3% > PLA 22.1% > CAF 20.7% |
| Mean power | 0.13 (−0.08, 0.34) | 0.13 (−0.01, 0.26) | 0.23 (−0.05, 0.50) | −0.00 (−0.23, 0.23) | 0.10 (−0.25, 0.45) | 0.10 (−0.21, 0.41) | MEN 79.1% > CHO 58.1% > CAF 55.7% > PLA 7.1% |
| Ratings of perceived exertion | −0.16 (−0.37, 0.06) | **−0.13 (−0.25, −0.02)** | 0.00 (−0.42, 0.42) | 0.02 (−0.20, 0.25) | 0.16 (−0.31, 0.63) | 0.13 (−0.30, 0.57) | CAF 75.0% > CHO 71.3% > MEN 34.1% > PLA 19.6% |

S7.2.2 Pairwise Meta-Analysis Results Assuming a Within-Participant Correlation Coefficient of r=0.70

| Outcome | Comparison | SMD (95% CI) | I^2^ (%) | P value |
| --- | --- | --- | --- | --- |
| Time to exhaustion | CHO vs PLA | **0.42 (0.16, 0.67)** | 0.00 | **<0.01** |
|  | CAF vs PLA | **0.56 (0.20, 0.92)** | 0.00 | **<0.01** |
|  | MEN vs PLA | **0.27 (0.04, 0.49)** | 0.00 | **0.02** |
| Work done during a fixed-time test | CHO vs PLA | **0.35 (0.16, 0.54)** | 0.00 | **<0.01** |
|  | CAF vs PLA | **0.55 (0.21, 0.89)** | 0.00 | **<0.01** |
|  | MEN vs PLA | 0.27 (−0.15, 0.70) | 30.47 | 0.20 |
| Time to complete a fixed amount of work | CHO vs PLA | **−0.13 (−0.24, −0.01)** | 0.00 | **0.03** |
|  | CAF vs PLA | 0.03 (−0.19, 0.24) | 0.00 | 0.82 |
|  | MEN vs PLA | −0.21 (−0.47, 0.06) | 0.00 | 0.13 |
| Mean power | CHO vs PLA | **0.14 (0.02, 0.25)** | 0.00 | **0.02** |
|  | CAF vs PLA | 0.14 (−0.29, 0.56) | 79.06 | 0.54 |
|  | MEN vs PLA | 0.23 (−0.02, 0.47) | 0.00 | 0.07 |
| Ratings of perceived exertion | CHO vs PLA | **−0.13 (−0.24, −0.02)** | 30.04 | **0.02** |
|  | CAF vs PLA | −0.15 (−0.50, 0.21) | 71.30 | 0.42 |
|  | MEN vs PLA | 0.00 (−0.32, 0.32) | 0.00 | 1.00 |
